# Discovery of KRB-456, a KRAS G12D Switch-I/II Allosteric Pocket Binder That Inhibits the Growth of Pancreatic Cancer Patient-derived Tumors

**DOI:** 10.1158/2767-9764.CRC-23-0222

**Published:** 2023-12-28

**Authors:** Aslamuzzaman Kazi, Alok Ranjan, Vasantha Kumar M.V., Bogos Agianian, Martin Garcia Chavez, Vignesh Vudatha, Rui Wang, Rajanikanth Vangipurapu, Liwei Chen, Perry Kennedy, Karthikeyan Subramanian, Jonathan C.K. Quirke, Francisca Beato, Patrick W. Underwood, Jason B. Fleming, Jose Trevino, Paul J. Hergenrother, Evripidis Gavathiotis, Said M. Sebti

**Affiliations:** 1 Department of Pharmacology and Toxicology and Massey Comprehensive Cancer Center, Virginia Commonwealth University, Richmond, Virginia.; 2 Department of Biochemistry, Department of Medicine, Montefiore Einstein Comprehensive Cancer Center, Albert Einstein College of Medicine, Bronx, New York.; 3 Department of Chemistry, Cancer Center at Illinois, University of Illinois at Urbana-Champaign, Urbana, Illinois.; 4 Department of Surgery, Virginia Commonwealth University, Richmond, Virginia.; 5 Drug Discovery Department, Moffitt Cancer Center, Tampa, Florida.; 6 Department of Gastrointestinal Oncology, Moffitt Cancer Center, Tampa, Florida.; 7 Department of Surgery, University of Florida, Gainesville, Florida.

## Abstract

Currently, there are no clinically approved drugs that directly thwart mutant KRAS G12D, a major driver of human cancer. Here, we report on the discovery of a small molecule, KRB-456, that binds KRAS G12D and inhibits the growth of pancreatic cancer patient-derived tumors. Protein nuclear magnetic resonance studies revealed that KRB-456 binds the GDP-bound and GCP-bound conformation of KRAS G12D by forming interactions with a dynamic allosteric binding pocket within the switch-I/II region. Isothermal titration calorimetry demonstrated that KRB-456 binds potently to KRAS G12D with 1.5-, 2-, and 6-fold higher affinity than to KRAS G12V, KRAS wild-type, and KRAS G12C, respectively. KRB-456 potently inhibits the binding of KRAS G12D to the RAS-binding domain (RBD) of RAF1 as demonstrated by GST-RBD pulldown and AlphaScreen assays. Treatment of KRAS G12D-harboring human pancreatic cancer cells with KRB-456 suppresses the cellular levels of KRAS bound to GTP and inhibits the binding of KRAS to RAF1. Importantly, KRB-456 inhibits P-MEK, P-AKT, and P-S6 levels *in vivo* and inhibits the growth of subcutaneous and orthotopic xenografts derived from patients with pancreatic cancer whose tumors harbor KRAS G12D and KRAS G12V and who relapsed after chemotherapy and radiotherapy. These results warrant further development of KRB-456 for pancreatic cancer.

**Significance:**

There are no clinically approved drugs directly abrogating mutant KRAS G12D. Here, we discovered a small molecule, KRB-456, that binds a dynamic allosteric binding pocket within the switch-I/II region of KRAS G12D. KRB-456 inhibits P-MEK, P-AKT, and P-S6 levels *in vivo* and inhibits the growth of subcutaneous and orthotopic xenografts derived from patients with pancreatic cancer. This discovery warrants further advanced preclinical and clinical studies in pancreatic cancer.

## Introduction

RAS proteins are guanine nucleotide-binding GTPases that serve as binary molecular switches that oscillate between their active GTP-bound and inactive GDP-bound forms to mediate fundamental cellular processes such as proliferation, differentiation, migration, and survival (1–3). These molecular switches are regulated by guanine nucleotide exchange factors (GEF) and GTPase-activating proteins (GAP; ref. 4). GAPs bind to active GTP-bound RAS proteins and stimulate their intrinsic GTPase activity to hydrolyze the GTP γ phosphate, leading to inactive GDP-bound RAS. When GEFs bind to GDP-RAS proteins, they lower their affinity for nucleotides, releasing GDP and allowing GTP to bind due to an excess of cellular GTP relative to GDP (4). Under normal physiologic conditions, this tightly regulated binary molecular switch mechanism allows the RAS proteins to transiently transduce biological signals, such as growth factors, mitogens, and cytokines, from outside to inside cells and regulates processes such as cell proliferation. For example, when the growth signal EGF binds its tyrosine kinase receptor EGFR, the receptor recruits GEFs such as SOS1, which catalyzes the exchange of GDP for GTP converting RAS to its active GTP-bound form. GTP-RAS can bind several effectors including RAF, PI3K, and RalGDS to activate the RAF/MEK/ERK, PI3K/AKT and RALGDS/RAL canonical pathways, eventually culminating into regulating cytosolic and nuclear events (5). In contrast, in human cancers, RAS often harbors mutations that lower RAS's affinity for GAPs, thus reducing its GTPase activity leading to persistently active GTP-bound RAS. This constitutive activation of RAS triggers pleiotropic oncogenic events, including uncontrolled proliferation, resistance to apoptosis, sustained angiogenesis, immune evasion, invasion, metastasis, and drug resistance (6, 7).

The three human *Ras* genes, *HRAS*, *KRAS*, and *NRAS*, are found frequently mutated in about one-third of human cancers, with KRAS mutations being the most prevalent among the three isoforms (∼86% of cases), and with NRAS and HRAS representing only 11% and 3%, respectively (8, 9). The frequency of KRAS mutations in patients with highly aggressive cancers such as pancreatic ductal adenocarcinoma (PDAC), colorectal cancer, and non–small cell lung carcinoma (NSCLC) is very high representing 90%, 35%, and 30% of all cases, respectively. The most common oncogenic KRAS mutations occur at amino acids involved in the GTPase active site such as glycine G12 and glutamine Q61, which lower the affinity of RAS for GAP and reduce the intrinsic GTPase activity of RAS, respectively, leading to a “GTP-locked” RAS (2, 10). In PDAC and colorectal cancer, the most common mutation is G12D followed by G12V, whereas in NSCLC, G12C is predominant (9). Tumors that harbor KRAS mutations are highly aggressive and are associated with poor prognosis and resistance to cancer therapies (11–13).

The high mutation frequency of KRAS and its pivotal role in driving human oncogenesis and resistance to cancer therapies prompted the search for pharmacologic agents to thwart mutant (mt) KRAS (14–16). The lack of well-defined targetable binding pockets on the KRAS protein has made it very challenging to design KRAS inhibitors for decades. However, recent breakthroughs have led to the identification of small molecules that bind covalently to mt KRAS G12C. These efforts were initially led by Shokat and colleagues (17) who used a thio-reactive warhead-containing chemical library and identified compounds that covalently attached to the cysteine 12 thiol group allowing the rest of the compound to bind under the switch-II region, a pocket only accessible in the GDP-bound form of KRAS G12C. Similar approaches have been used to covalently target the cysteine of KRAS G12C, and two of the resulting drugs, sotorasib and adagrasib, have now been FDA approved for the treatment of patients with advanced NSCLC whose tumors harbor KRAS G12C mutations (18–23). In addition to the KRAS G12C binders, other compounds have been identified that bind other isoforms of KRAS, including recently MRTX1133 that binds KRAS G12D (24–29). In this article, we report on the discovery of a small molecule, KRB-456, that binds to KRAS G12D by forming interactions with a dynamic allosteric binding pocket within the switch-I/II region. KRB-456 binds to KRAS G12D, KRAS wild-type (WT), KRAS G12V, and KRAS G12C with dissociation constants of 247, 483, 392, and 1,410 nmol/L, respectively. KRB-456 suppresses the cellular levels of KRAS bound to GTP, and inhibits binding of KRAS to RAF1 and signaling pathways downstream of RAS. Importantly, KRB-456 is effective at thwarting the *in vivo* growth of subcutaneous and orthotopic tumors from pancreatic cancer patients with mt KRAS G12D and G12V who have relapsed after chemotherapy and radiotherapy.

## Materials and Methods

### Cell Lines, Cell Culture, and Reagents

Human pancreatic cancer cell lines Panc0203, Panc1, Panc0403, Capan1, MiaPaCa2, and BxPC3 were obtained from the ATCC. Panc0203, Panc0403, Capan1, and BxPC3 cells were cultured in RPMI1640 whereas Panc1 and MiaPaCa2 cells were cultured in DMEM (Gibco, Thermo Fisher Scientific). All culture media were supplemented with 10% heat-inactivated FBS (R&D Systems), and 1% penicillin-streptomycin (Sigma). Cells were grown at 37°C with 5% CO_2_. Cell lines were used between passage 3 and 5 after they were taken out of the liquid nitrogen. Cell lines were authenticated by the University of Arizona Genetics Core. All cell lines were *Mycoplasma* free, monitored regularly with HEK-blue2 cells and Mycoplasma Detection Kit from InvivoGen (catalog no. rep-pt1).

### Chemical Library Synthesis

The natural product-inspired small-molecule synthetic chemical library of 560 chemically diverse compounds with high structural and stereochemical complexity was synthesized as described previously (30–34).

### KRAS G12D/GST-RBD Alpha Screen

GTP-loaded biotinylated-KRAS G12D (2-188) was added to the wells of 384-well plates (OptiPlate-384; PerkinElmer) at a final concentration of 250 nmol/L in 50 mmol/L Tris (pH 7.4), 150 mmol/L NaCl, 5 mmol/L MgCl_2_, and 0.1 mmol/L DTT buffer. The 560 compounds from the chemical library (30) were added in a one-well-one compound format (10 µmol/L final concentration). After a 5-minute incubation at room temperature, GST-RBD was added (250 nmol/L final concentration). After incubation at room temperature shaking at 700 rpm for 1 hour, a mixture of streptavidin-coated alpha donor beads (binds biotinylated-KRAS) and glutathione-coated alpha acceptor beads (binds Raf1-GST-RBD) were added [the beads were from PerkinElmer and were in 50 mmol/L Tris (pH 7.4), 0.01% Tween20]. The plates were then spun at 500 rpm for 1 minute, and incubated at room temperature while shaking at 700 rpm for 1 hour. The luminescence was then determined at an excitation at 680 nm with an emission of 520–620 nm, using an alpha-compatible reader (BioTek Synergy Neo2 Plate Reader). All steps were performed under passive light to prevent photobleaching of the beads.

### KRAS G12D/GST-RBD Pulldown Assay

For pulldown experiment, KRAS G12D was preloaded with either GTP or GDP as described above. GDP- or GTP-bound KRAS G12D proteins (250 nmol/L in 100 µL) were then incubated with indicated compound at 37°C for 15–16 minutes. GST-RBD (final 500 nmol/L) was then added and the mixture rocked at 37°C for 1 hour. Pierce glutathione agarose beads (Thermo Fisher Scientific; 20 µL) were then added and the mixture rocked at 37°C for 1 hour. The beads were then washed and the samples were processed for SDS-PAGE and Western blotting with respective antibodies to check the interaction between KRAS-GTP and GST-RBD proteins in presence or absence of the compounds. All the protein dilutions and washes were carried out in a buffer containing 25 mmol/L Tris (pH 7.5), 100 mmol/L NaCl, 5 mmol/L MgCl_2_, 1% NP-40, 5% glycerol, and 0.1 mmol/L DTT.

### Isothermal Titration Calorimetry

All isothermal titration calorimetry (ITC) experiments were carried out at 25°C on a MicroCal PEAQ ITC Automated (Malvern). The ITC titrations were conducted in forward fashion with 2 mmol/L KRB-456 in the syringe and 100 µmol/L protein in the sample cell. The final ITC buffer in the syringe and the cell was 25 mmol/L Tris HCl (pH 8.0), 100 mmol/L NaCl, 0.1 mmol/L DTT, 5% glycerol, 1% NP40, 5 mmol/L MgCl_2_, and 5% DMSO. ITC titration experiments were carried out with 19 injections, 2 µL per injection, and 150 seconds spacing between each injection. Reference cell power was set to 5 µcal/second and stirring speed was 750 rpm. Data were analyzed using a one site binding model with the Malvern analysis software. All ITC experiments were performed at the biophysical facility at the Institute for Structural Biology, Drug Discovery and Development (ISB3D) at Virginia Commonwealth University (Richmond, VA) and the Massey Cancer Center.

### Nuclear Magnetic Resonance Spectroscopy

The uniformly ^15^N-labeled KRAS-G12D^GDP^ protein samples were prepared by growing the bacteria in a minimal medium as described previously. Nuclear magnetic resonance (NMR) buffer: 1X PBS (pH 7.4), 1 mmol/L MgCl_2_, and 1 mmol/L DTT buffer with 10% D_2_O used to prepare a ^15^N-labeled sample for NMR measurements. The NMR experiments were acquired using a Bruker 600 MHz NMR spectrometer equipped with a 5 mm H/F-TCI cryoprobe capable of applying pulse field gradient along the *Z*-axis. All experiments were performed at 298 K, using independent samples for each experimental measurement as a 70 or 400 µL sample volume in a 1.7-mm or 5-mm Shigemi NMR tube, respectively. All samples were DMSO matched with regular 5% DMSO. ^1^H-^15^N heteronuclear single quantum coherence (HSQC) correlation spectra were recorded on ^15^N-labeled KRAS-G12D^GDP^ at 50 µmol/L concentration in the presence and absence of 200 µmol/L of KRB-456 and IIA-15D compounds. Furthermore, the KRAS-G12D^GDP^ sample from a fresh batch was subjected to nucleotide exchange from GDP to GCP as described above. A new set of ^1^H-^15^N HSQC correlation spectra were recorded on ^15^N-labeled KRAS-G12D^GCP^ at 50 µmol/L concentration in the presence and absence of 200 µmol/L of KRB-456 compound. All the ^1^H-^15^N HSQC experiments acquired with 1.1 second recycle delay, 48 scans with 2048*160 [NUS Amount 50%, NUS Points 40, and NUS ^15^NT2(s) 0.1 ms] points in direct/indirect dimensions, respectively. The NMR spectra processing and reconstruction were achieved using NMRPipe (35) SMILE (Sparse Multidimensional Iterative Lineshape-Enhanced) method (36) and spectral analysis accomplished with NMRFAM-SPARKY (37). The BMRB (Entry code: 27719/28022) deposited ^1^H-^15^N chemical shift values were used to obtain the ^1^H-^15^N backbone assignments of KRAS-G12D^(GDP or GCP)^. The weighted average chemical shift perturbation (CSP) calculated using equation CSP = √(Δδ^1^H)^2^+(Δδ^15^N/5)^2^ (in p.p.m). The absence of a bar in the CSP plot indicates no chemical shift difference or the presence of proline residue or a residue that overlaps with other residue or is missing and therefore not used in the analysis. The significance threshold for backbone amide proton's chemical shift change was calculated on the basis of the average chemical shift across all residues plus one STDev (38).

### KRAS Pulldown from Whole-cell Lysates Using RAF1-GST-RBD

Panc0203 and Panc1 human pancreatic cancer cells were treated with DMSO control or 20 µmol/L KRB-456 for 15 minutes. Cells were then washed twice with ice-cold TBS followed by addition of lysis buffer [25 mmol/L Tris (pH 7.5), 150 mmol/L NaCl, 5 mmol/L MgCl_2_, 1% NP-40, 5% glycerol, and protease and phosphatase inhibitor cocktail tablet (Thermo Fisher Scientific)]. Cell lysates were centrifuged and the supernatants were collected for GST-RBD pulldown as follows. Lysate supernatants from Panc0203 and Panc1 were added to 50 µg of GST-RBD and 100 µL of glutathione agarose beads (Thermo Fisher Scientific) in a total volume of 1 mL, and the mixtures were then rocked at 4˚C for 2 hours. After incubation beads were washed three times with lysis buffer and boiled at 100°C for 10 minutes with 4X Laemmli sample buffer (Bio-Rad) and analyzed by Western blotting with respective antibodies.

### Co-immunoprecipitation

Human pancreatic cancer cell lines Panc0203 and Panc1 were treated with DMSO control or 20 µmol/L KRB456 for 15 minutes. Cells were then washed twice with ice-cold PBS, followed by addition of lysis buffer [25 mmol/L Tris (pH 7.5), 150 mmol/L NaCl, 1 mmol/L EDTA (pH 8.0), 1% NP-40, 5% glycerol and protease and phosphatase inhibitor cocktail tablet (Thermo Fisher Scientific)]. Cell lysates were centrifuged, and the lysate supernatants from Panc0203 (1 mg) and Panc1 (1 mg) were precleared using Protein A/G PLUS-Agarose beads (Santa Cruz Biotechnology), for 30 minutes at 4°C, and 30 µg RAS antibody (Abcam, catalog no. ab180772) was then added to the precleared supernatants and the mixture rocked overnight at 4°C. Separate normal rabbit IgG (EMD Millipore, catalog no. 12-370) mixtures were used as internal control. After incubation, immunoprecipitates were collected by adding 30 µL of Protein A/G PLUS-Agarose beads and rocking the mixtures for 4 hours at 4°C. After incubation beads were washed four times with lysis buffer and boiled at 100°C for 10 minutes with 4X Laemmli sample buffer (Bio-Rad) and analyzed by Western blotting as described below.

### Western Blot Analysis

To prepare whole-cell lysates, cells were washed twice with cold PBS and lysed in M-PER Mammalian Protein Extraction Reagent (product no. 78501, Thermo Fisher Scientific) supplemented with Pierce protease and phosphatase inhibitor cocktail (product no. A32961, Thermo Fisher Scientific). Tumor tissue samples were lysed in T-PER Tissue Protein Extraction Reagent (product no. 78510, Thermo Fisher Scientific) with above supplement. The automatic hand-operated OMNI-TIP Homogenizer (Omni International, Inc.) was used to homogenize the tumor tissues. Lysates from whole cells and tumor homogenates were then processed for SDS-PAGE Western blotting. The following antibodies were used: Phospho-MEK1/2 (S217/221; catalog no. 9154S), MEK1/2 (catalog no. 4694S), Phospho-AKT (S473; catalog no. 9271S), Phospho-AKT (T308; catalog no. 4056S), AKT (catalog no. 9272S), Phospho-Erk1/2 (catalog no. 4370S), Erk1/2 (catalog no. 4695S), Phospho-S6 Ribosomal Protein (Ser 235/236; catalog no. 4858S), S6 Ribosomal Protein (catalog no.2217S), cleaved-CASP-3 (catalog no. 9664), cleaved-PARP (catalog no. 9541) from Cell Signaling Technology; anti-β-ACTIN (catalog no A5441-.2ML from Sigma-Aldrich; KRAS (catalog no. OP24, from EMDMillipore), and RAF1 (catalog no. sc-7267, Santa Cruz Biotechnology), horseradish peroxidase–conjugated goat anti-GST antibody (catalog no. A190-121P from Bethyl Laboratories).

### Cell Viability Assay

Cell viability assays were carried out using the CellTiter-Glo Luminescent Cell Viability Assay (Promega). Briefly, cells were seeded in 96-well plates at a density of 2,000 cells/well, allowed to adhere overnight, and treated with DMSO control or different doses of KRB-456 for 72 hours, after which they were processed for viability using CellTiter-Glo reagent. All the experiments were performed in triplicate and each condition was performed in replicates of 3–6 wells.

### Antitumor Efficacy Studies of Subcutaneously Implanted Patient-derived Xenografts from Patients with Pancreatic Cancer

We first determine which KRB-456 doses are tolerable in NSG mice. To this end, healthy NSG mice were treated (3 mice per treatment group) daily intraperitoneally for up to 21 days either with vehicle [10% DMSO, 20% propylene glycol, and 28% of 2-Hydroxypropyl-β-cyclodextrin (HPCD; 40% stock in dH_2_O)] or KRB-456 (4, 5, or 10 mpk KRB-456 dissolved in the same vehicle). An additional group of 3 mice were not treated and used as control for the vehicle itself. KRB-456 at 10 mpk/day was not tolerable and mice started losing weight on day 2 and dying on day 8. In contrast, none of the mice treated with vehicle, 4 or 5 mpk died. Throughout the 21 days, the average % change in mouse weights in the control non-treated mice varied between −3% to +4%, in the vehicle-treated mice between −1% to +3%, in the 4 mpk between −6% to +3% and in 5 mpk-treated mice between −6% to 0%. As such, we used 4 and 5 mpk for the antitumor patient-derived xenograft (PDX) studies described below, and found the following changes in mouse weights. The average % change in mouse weights in patient G148 PDX-bearing mice treated with vehicle varied between 0% to +8%, and treated with 5 mpk KRB-456 between 0% to −10%. The average % change in mouse weights in patient G166 PDX-bearing mice treated with vehicle varied between −7% to +1%, and treated with 5 mpk KRB-456 between −11% to −1%. The average % change in mouse weights in patient G174 PDX-bearing mice treated with vehicle varied between −5% to 0%, and treated with 5 mpk KRB-456 between −13% to +5%. The average % change in mouse weights in patient G160 PDX-bearing mice treated with vehicle varied between −6% to +1%, and treated with 4 mpk KRB-456 between −10% to +1%. For the orthotopic patient G166 PDX, the average % change in mouse weights patient G166 PDX-bearing mice treated with vehicle varied between 0% to +4%, and treated with KRB-456 between 0% to −8%.

To assess the antitumor potential of KRB-456 in PDXs, we obtained fresh tumor biopsies from 4 patients with pancreatic cancer with KRAS mutation [University of Florida, Institutional Review Board (IRB) protocol 201600873]. Written consent was obtained from the subjects, and the research was conducted according to International Ethical Guidelines for Biomedical Research Involving Human Subjects. The fresh tumor biopsies were from the following 4 patients: Patient G148 was a 79-year-old female who underwent a radical antegrade modular pancreatosplenectomy. Pathology revealed a 4.5 cm, moderately differentiated, ductal adenocarcinoma with lymphovascular and perineural invasion, 0 out of 10 involved lymph nodes, and negative surgical margins (R0). Pathologic staging was T3N0. KRAS mutation was G12D. She completed four cycles (day 1, 8, and 15) of adjuvant gemcitabine over 6 months and radiotherapy. She had evidence of recurrence 10 months after her last cycle of chemotherapy. Patient G166 was a 50-year-old male who underwent a laparoscopic pancreaticoduodenectomy. Pathology revealed indeterminate size (greater than 2 cm but less than 4 cm), moderately differentiated, ductal adenocarcinoma with lymphovascular and perineural invasion, 1 out of 28 involved lymph nodes, and positive surgical margins (R1). Pathologic staging was T2N1. KRAS mutation was G12D. He completed eight cycles of adjuvant gemcitabine/capecitabine over 5 months. He had no evidence of disease 17 months after his last cycle of chemotherapy. Patient G174 was a 63-year-old female who underwent a distal pancreatectomy and splenectomy. Pathology revealed a 4.3 cm, poorly differentiated, adenosquamous carcinoma with lymphovascular and perineural invasion, 1 out of 21 involved lymph nodes, and positive surgical margins (R1). Pathologic staging was T3N1. KRAS mutation was G12V. She had a presumed recurrence 6 weeks after surgery and was deceased 8 weeks after surgery without receiving adjuvant therapy. Patient G160 was a 63-year-old male who underwent a laparoscopic pancreaticoduodenectomy. Pathology revealed a 1.5 cm, poorly differentiated, ductal adenocarcinoma with lymphovascular and perineural invasion, 3 out of 24 involved lymph nodes, and negative surgical margins (R0). Pathologic staging was T1N1. KRAS mutation was G12D. He completed five cycles of 5-fluorouracil (5-FU) with radiotherapy over 5 weeks followed by 6 cycles of adjuvant gemcitabine with capecitabine over 5 months (capecitabine stopped after 2 cycles for rash). He had evidence of recurrence 8 months after his last cycle of chemotherapy.

The mice were housed, maintained, and treated, and all the experiments were performed under protocols approved by the Moffitt Cancer Center, University of South Florida (protocol no. R IS00006177), University of Florida (protocol no. 201406590), and Virginia Commonwealth University (protocol no. AD10002523) Institutional Animal Care and Use Committees according to federal, state, and institutional guidelines and regulations. Upon pancreatic tumor resection, fresh 2-mm tumor pieces were taken on ice to the animal surgery suite for subcutaneous implantation into NOD.Cg-Prkdcscid Il2rgtm1Wjl/SzJ (NSG) mice. A viable tumor piece was placed in the right flank subcutaneous tissue of anesthetized mice and the skin was closed (generation 1). Once tumors reached endpoint (1.5 cm in diameter), tumors were divided evenly into 2–4 mm pieces and reimplanted into NSG mice as above (generation 2). Generation 3 was generated similarly (39).

When the tumor volumes from generation 3 reached approximately 200 mm^3^, the mice were randomized into two groups, vehicle (10% DMSO, 20% propylene glycol, and 28% of HPCD, and KRB-456 reconstituted in the same vehicle. Patient G148 PDX mice were injected intraperitoneal injection daily for 24 days with vehicle (*n* = 6) or 5 mg/kg (mpk)/day KRB-456 (*n* = 6; except for no treatments for days 12 and 13). Patient G166 PDX mice were injected intraperitoneally with vehicle (*n* = 7) and 5 mpk/day KRB-456 (*n* = 6) with intermittent cycles of 7 days of treatment followed by 3 days of no treatment for a total of 24 days. Patient G174 PDX mice were injected intraperitoneally with vehicle (*n* = 6) and 5 mpk/day KRB-456 (*n* = 6) daily for 17 days except for days 11, 12, and 14 to 17 with no treatment. Patient G160 PDX mice were injected intraperitoneally with vehicle (*n* = 10) and 4 mpk/day KRB-456 (*n* = 10) with intermittent cycles of 5 days of treatment followed by 2 days of no treatment for a total of 27 days. The tumors were measured with an electronic caliper three times per week. Tumor volume was calculated using the following formula: volume = (*L*^2^*W*)/2, where *L* is length and *W* is width, with width defined as the largest measurement and length is the smallest measurement.

### Antitumor Efficacy Studies of Orthotopically Implanted PDXs from Patients with Pancreatic Cancer

Human pancreatic cancer specimens (patient G160) were initially implanted subcutaneously for tumor expansion as described above. Upon reaching an endpoint of 1.5 cm in maximum diameter, the tumors were harvested and divided into 3–4 mm pieces. Orthotopic implantation was then performed in NSG mice as described by us previously (40). On day 20 after tumor implantation, the mice were injected daily intraperitoneally with either vehicle (*n* = 7) or 5 mpk KRB-456 (*n* = 7) for 30 days. Tumors were followed via abdominal ultrasound twice a week using the LOGIQ e NextGen Ultrasound machine (ANTECH Sound Imaging). At each timepoint, the mice were placed under appropriate anesthesia and the abdomen was saved thoroughly. Ultrasound was utilized to identify key structures including left kidney, stomach, liver, and bladder. Once these structures were noted, we identified the tumor. Where possible, the tumor and left kidney were encompassed in the same image. However, this could not be performed in all instances depending upon the axial location of the tumor. Scanning was performed using an 18 MHz linear probe and measurements were taken. Tumors were followed by ultrasound starting on day 40, but only became big enough to be measurable starting on day 54 after implantation. Tumor measurements continued until the experiment was terminated on day 97 after implantation.

### Effects of KRB-456 on Viability in Two- and Three-dimensional Cocultures of Low-passage Primary and Metastatic mt KRAS Adenocarcinoma Cells Derived from Patients with Pancreatic Cancer

Low-passage (<20) human pancreatic cancer cell lines were derived from 8 patients with pancreatic cancer using IRB-approved (MDA Cancer Center protocol LAB07-0854) and previously described methods (41–43). The cell lines were provided to Dr. Jason B. Fleming by Dr. Michael P. Kim (MD Anderson Cancer Center). Written consent was obtained from the subjects, and the research was conducted according to Declaration of Helsinki. The cells were plated at 3,000/well in triplicate in a 96-well flat-bottom plate in RPMI1640 medium with 10% FBS. Cells were subsequently treated for 72 hours with DMSO or KRB-456 at 3, 10, and 30 µmol/L. For three-dimensional (3D) cultures, cold Matrigel growth factor–reduced, phenol red-free solution was added to wells, spread evenly, and allowed to incubate at 37°C for 30 minutes to solidify. Pancreatic cancer cells (3,000/well) were resuspended in RPMI-2% Matrigel medium-10% FBS overlaid on the solidified Matrigel and cultured for 24 hours before treatment. For 3D coculture with pancreatic stellate cells, human pancreatic stellate cells were grown until confluent in RPMI1640 with 10% FBS. Matrigel was added as in 3D culture and pancreatic cancer cells and human pancreatic stellate cells were resuspended at 3,000 cells/well in RPMI-2% Matrigel medium supplemented with 10% FBS, overlaid at 1:1 ratio, and cultured for 24 hours before treatment. Cell viability was determined by the CellTiter-Glo luminescent cell viability assay (Promega) according to the manufacturer's protocol. Briefly, cells (10^3^ cells/well) were seeded in 384-well plates, allowed to adhere overnight, and treated with vehicle (DMSO) or KRB-456 for 72 hours, after which they were processed for viability using CellTiter-Glo reagent. Data were normalized to percentage of control, and IC_50_ values were determined. IC_50_ was calculated using GraphPad Prism 7.02 software. Each condition was performed in replicates of 6 wells. Live-cell imaging was carried out with the IncuCyte S3 live-cell imaging system (Essen Bioscience). Analysis was performed using the basic analyzer module within the IncuCyte S3 2018B software to determine cell growth confluency and day 0 scan was used as control.

### Effects of KRB-456 Treatment of Mice on the Levels of Tumor P-MEK, P-ERK, P-AKT, P-S6, CASPASE 3, and PARP in Xenografts from Patients with Pancreatic Cancer

Fresh tumor biopsies from patient G160 were implanted subcutaneously in NSG mice as described above, and when the average tumor volume reached 200 mm^3^, the mice were randomized and treated either with vehicle (3 mice) or 5 mpk KRB-456 (3 mice). Two hours after treatment, tumors were harvested and lysates were processed for Western blotting as described above. Densitometric analysis of Western blots was performed to determine % change or fold increase of specific protein after KRB-456 treatment.

### Data Availability Statement

The datasets generated during and/or analyzed during the current study are available on reasonable request.

## Results

### Identification of KRAS Binder-456 and its Analogs as Disruptors of the Binding of GTP-KRAS G12D to the RAS-binding Domain of RAF1

**FIGURE 1:**
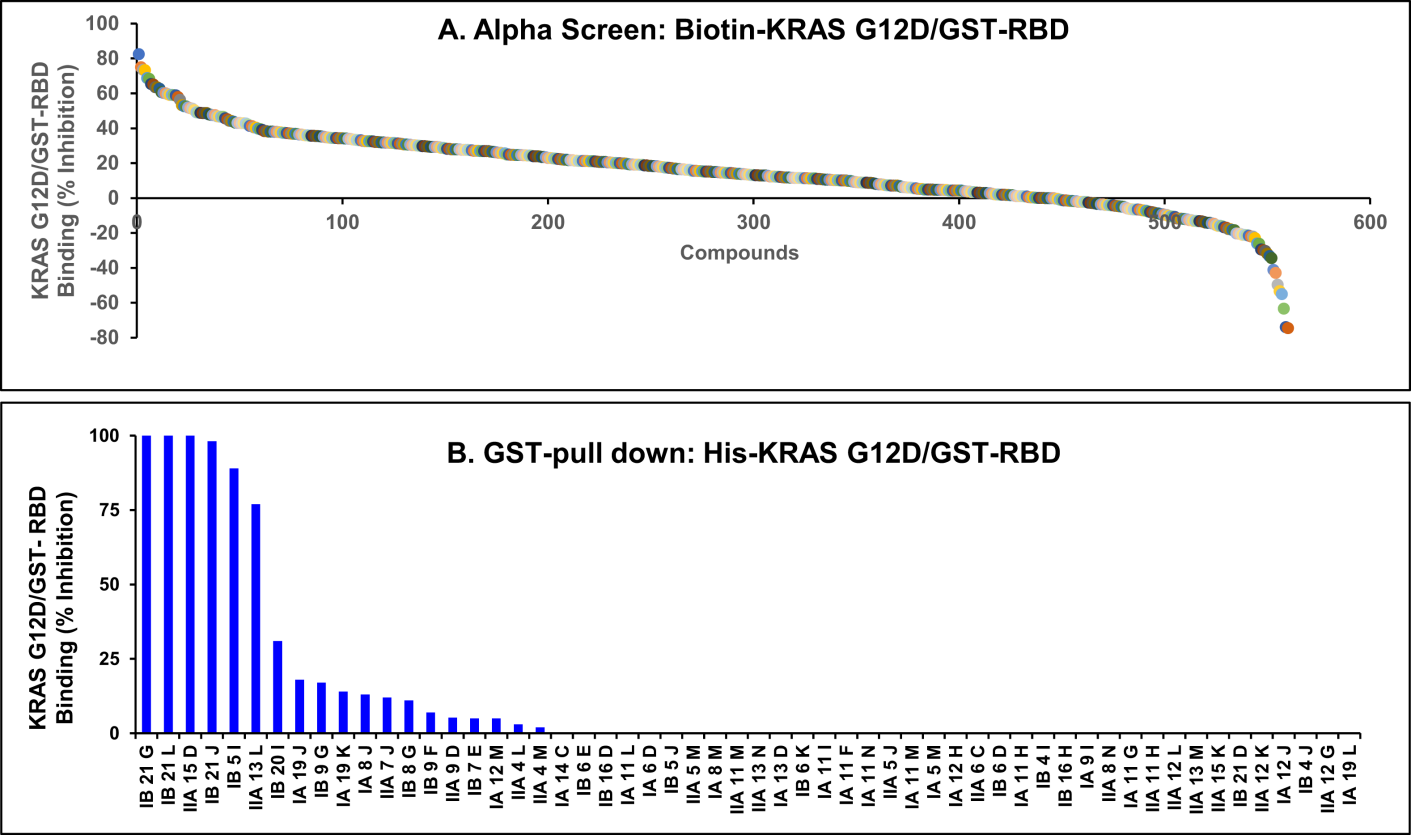
Alpha screen and GST pulldown assays identify disruptors of the binding of mt KRAS G12D to the RBD of RAF1. **A,** Alpha screen was used in a one-compound (10 µmol/L)-one well 384-well plate format to identify KRAS G12D/RBD binding disruptors from a natural product-inspired 560 synthetic compound library. The compounds were ranked from highest to lowest based on % inhibition of the KRAS G12D/RBD binding. **B,** The top ranked 56 hits (10% of the 560-compound library) from the alpha screen were selected for confirmation by a GST pulldown assay, and six of these were found to inhibit the binding of KRAS G12D to GST-RBD potently (77% to 100%) whereas the remaining 50 compounds weakly inhibited the interaction (0% to 31%).

**FIGURE 2:**
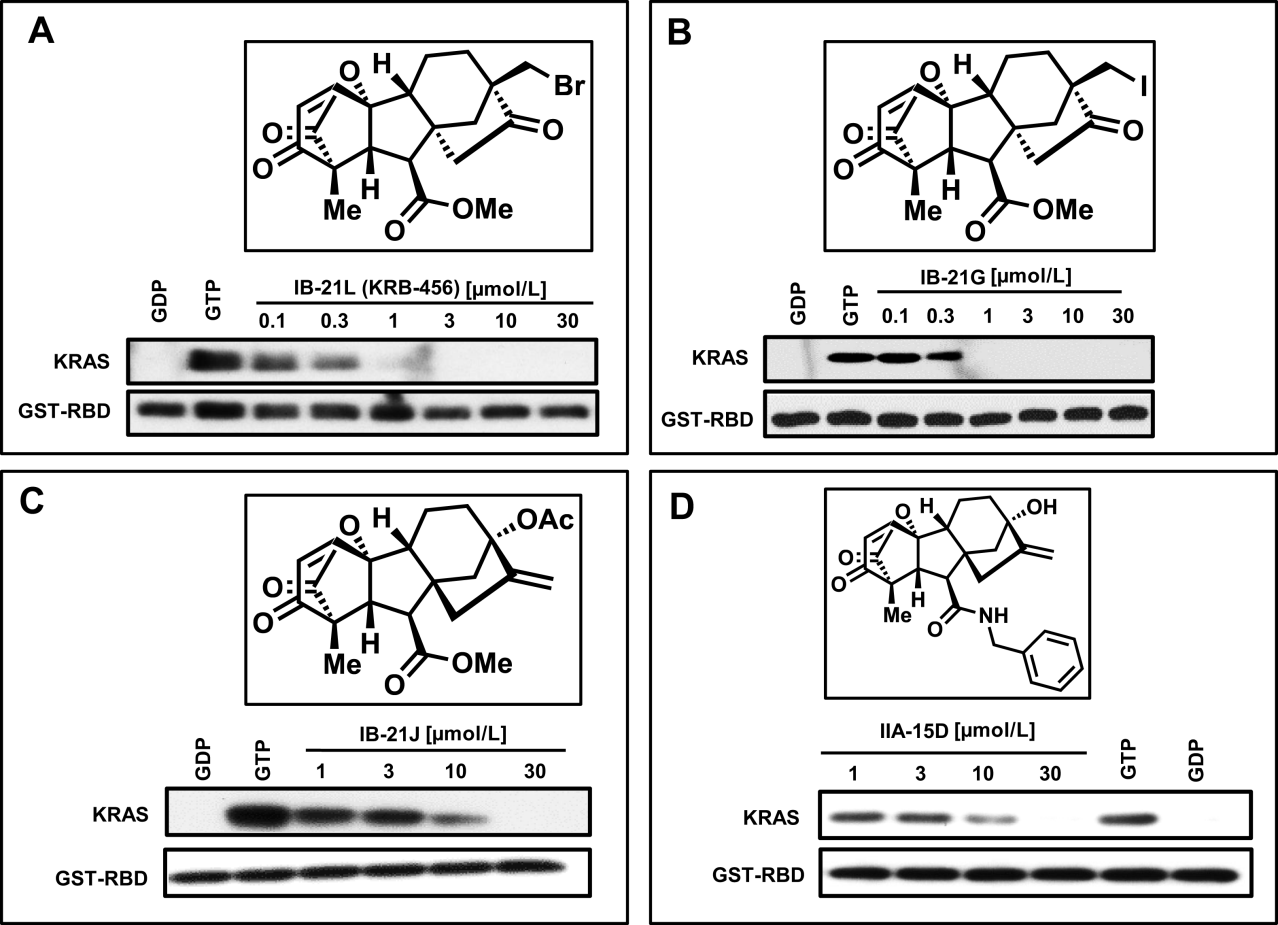
SAR studies of the inhibition of KRAS G12D/GST-RBD binding. The top four confirmed hits from Fig. 1 were all derivatives of gibberellic acid and were evaluated for their potency to inhibit GTP-KRAS G12D/GST-RBD binding by GST pulldown assays. Concentration–response experiments identified IB-21 L (KRB-456; IC_50_ = 0.26 µmol/L) as the most potent compound followed by IB-21G (IC_50_ = 0.36 µmol/L), IB-21J (IC_50_ = 2.02 µmol/L), and IIA-15D (IC_50_ = 10.82 µmol/L).

In an effort to discover small molecules that bind mt KRAS G12D, we first used an AlphaScreen assay to identify disruptors of the binding of biotin-tagged KRAS G12D to GST-tagged RBD (RAS-binding domain of RAF1) from a natural product-inspired synthetic chemical library of 560 chemically diverse small molecules with high structural and stereochemical complexity (30). The 560 compounds were screened at 10 µmol/L in 384-well plates using a “one well-one compound” format. Figure 1A shows the % inhibition of GTP-KRAS G12D binding to RBD with the most potent compound inhibiting this interaction by 83%. The top 10% high ranking compounds (56 of the 560 compound library) inhibited the KRAS G12D/RBD interaction between 83% and 41% and were selected for confirmation by a GST pulldown secondary assay using His-tagged-KRAS G12D and GST-RBD. Figure 1B shows that out of the top 56 selected compounds from the AlphaScreen, 6 (IB-21G, IB-21L, IIA-15D, IB-21J, IB-5I, and IIA-13L) inhibited the binding of GTP-KRAS G12D to GST-RBD potently (100% to 77% inhibition) whereas the remaining 50 compounds only weakly inhibited the interaction (0% to 31%). Furthermore, among the six most potent compounds, 4 (IB-21G, IB-21L, IIA-15D, and IB-21J) share the same pharmacophore (all gibberellic acid derivatives; Fig. 2) and were most potent at inhibiting the KRAS G12D binding to GST-RBD by 98% to 100% (Fig. 1B). The other two compounds, IB-5I and IIA-13L, have different pharmacophores and inhibited the interaction by 89% and 77%, respectively (Fig. 1B).

We next focused on the four gibberellic acid derivatives and determined their IC_50_ values to gain a better understanding of the structure-activity relationship (SAR) for their inhibition of the binding of KRAS G12D to GST-RBD using GST pulldown assays. Figure 2 shows that, in the absence of compounds, GST-RBD binds KRAS G12D when bound to GTP but not to GDP. Figure 2 also shows that the binding of GTP-bound KRAS G12D to GST-RBD is inhibited in a concentration-dependent manner by all four gibberellic acid derivatives. Compound IB-21 L [referred to herein as KRAS binder-456 (KRB-456)] was the most potent derivative and inhibited the KRAS G12D/GST-RBD interaction with an IC_50_ value of 260 nmol/L (Fig. 2A; Supplementary Table S1A). Substituting the bromomethyl of KRB-456 with an iodomethyl as in IB-21G slightly decreased the potency (IC_50_ value of 360 nmol/L; Fig. 2B; Supplementary Table S1A), suggesting that halide substitution is tolerable. However, replacing the bromomethyl of KRB-456 with an acetate group and the adjacent carbonyl with an exocyclic olefin, and inducing a stereochemical inversion as in IB-21J, resulted in a significant loss of potency by about 10-fold (IC_50_ value of 2,020 nmol/L; Fig. 2C; Supplementary Table S1B). Furthermore, exchanging the methyl ester and the acetate of IB-21J with benzylamide and hydroxyl, respectively, as in IIA-15D, is not tolerable and resulted in significant loss of potency to inhibit the binding of GTP-KRAS G12D to GST-RBD (IC_50_ value of 10,820 nmol/L; Fig. 2D; Supplementary Table S1B).

**FIGURE 3:**
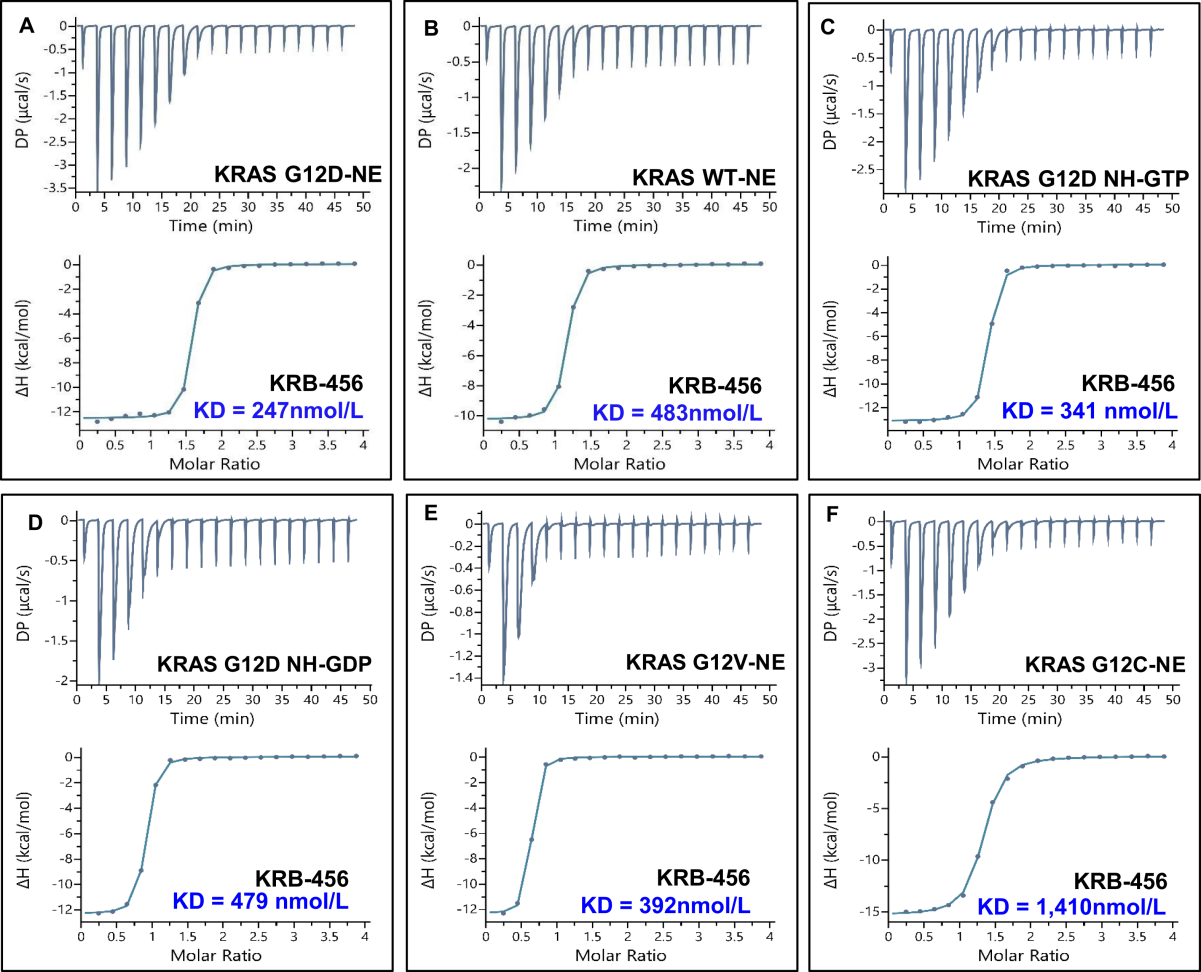
Determination of binding affinities of KRB-456 for KRAS G12D-NE, KRAS G12D NH-GTP, KRAS G12D NH-GDP, KRAS WT-NE, KRAS G12V-NE, and KRAS G12C-NE using ITC. ITC assays were performed using 2 mmol/L KRB-456 in the ITC injector and 100 µmol/L protein in the ITC sample cell as described in Materials and Methods.

We next determined whether KRB-456 binds directly to KRAS G12D using ITC. Figure 3A shows that KRB-456 binds KRAS G12D with a dissociation constant Kd = 247 nmol/L. Consistent with the GST pulldown results of Fig. 2 and Supplementary Table S1, ITC data show that KRB-456 analogs IB-21G (Kd = 641 nmol/L; Supplementary Fig. S1A), IB-21J (Kd = 4,810 nmol/L; Supplementary Fig. S1B) and IIA-15D (Kd = 11,600 nmol/L; Supplementary Fig. S1C) bind KRAS G12D with lower affinity.

### KRB-456 Binds to KRAS G12D with 1.5-, 2-, and 6-fold Higher Affinity Than to KRAS G12V, KRAS WT, and KRAS G12C, Respectively

Next, we compared the binding affinity of KRB-456 with KRAS G12D, KRAS G12V and KRAS G12C mutant isoforms as well as WT KRAS. KRB-456 bound mt KRAS G12D (Kd = 247 nmol/L; Fig. 3A) with about 2-fold higher affinity than WT KRAS (Kd = 483 nmol/L; Fig. 3B) and about 1.5-fold higher affinity than KRAS G12V (Kd = 392 nmol/L; Fig. 3E). KRB-456 bound mt KRAS G12D with about 6-fold higher affinity than KRAS G12C (Kd = 1,410 nmol/L; Fig. 3F).

All the ITC experiments described above were done with KRAS proteins that were non-exchanged (NE). To compare the binding affinity of KRB-456 with GTP- versus GDP-bound KRAS G12D, we performed nucleotide exchange with non-hydrolysable analogs of GDP (NH-GDP) and of GTP (NH-GTP) as described in the Materials and Methods section. KRB-456 binds to KRAS G12D-NE (Kd = 247 nmol/L; Fig. 3A) with about 1.4-fold higher affinity than to NH-GTP-loaded KRAS G12D (Kd = 341 nmol/L; Fig. 3C) and about 2-fold higher affinity than to NH-GDP–loaded KRAS G12D (Kd = 479 nmol/L; Fig. 3D).

### KRB-456 Binds to KRAS G12D at a Switch-I/II Allosteric Pocket

**FIGURE 4:**
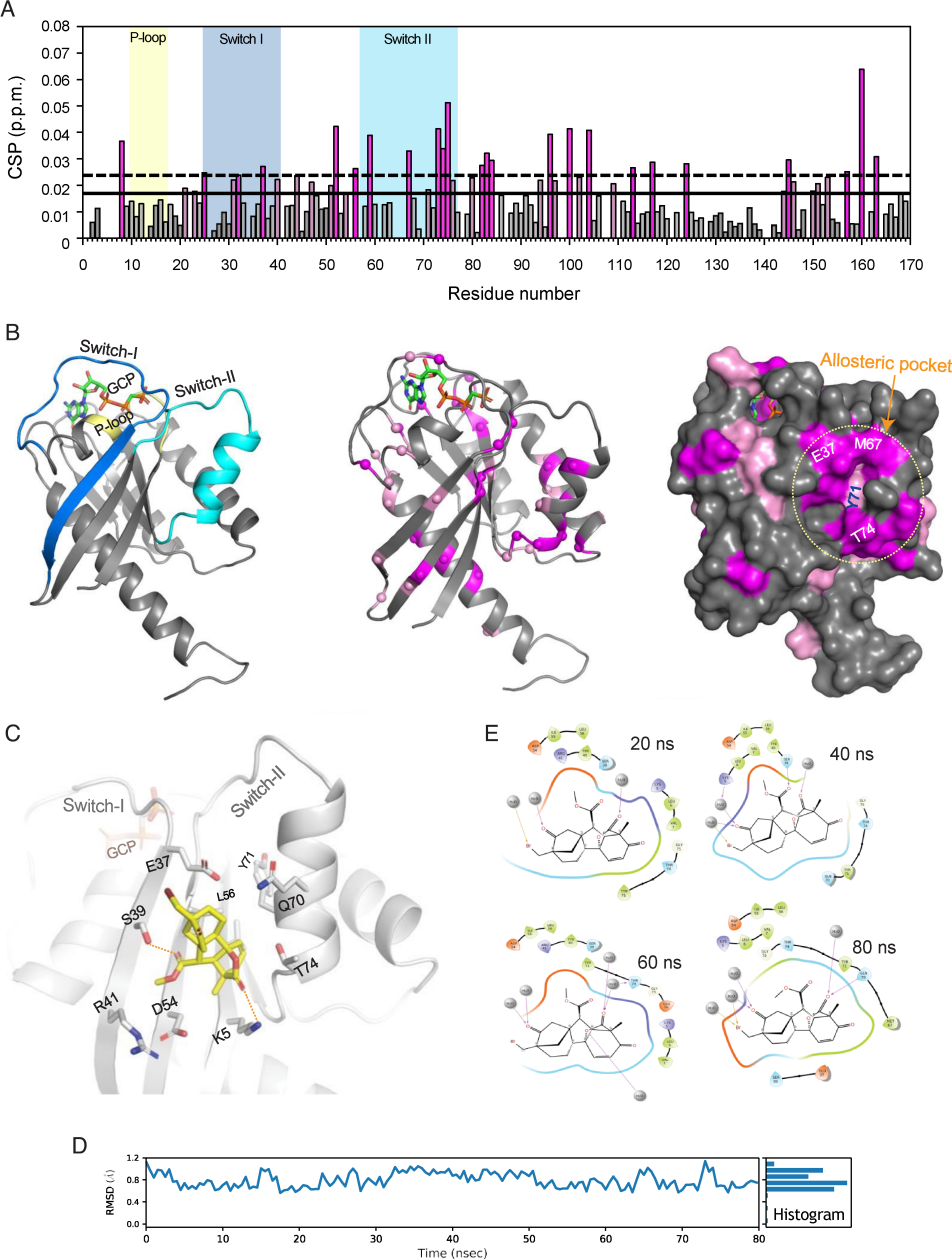
KRB-456 binds to KRAS G12D^GDP^ at a switch-I/II allosteric site. **A,** Measured CSPs of ^15^N-labeled KRAS G12D^GDP^ upon KRB-456 titration up to a ratio of 1:4 KRAS G12D^GDP^: KRB-456 are plotted as a function of KRAS residue number. Residues with chemical shift ranges over the significance threshold or 1.5 times the threshold are colored pink and magenta, respectively. Residues associated with the P-loop, the switch-I and switch-II structural motifs are indicated with yellow, blue and cyan shading, respectively. Data are representative of three independent experiments. **B,** Ribbon representation of KRAS G12D^GCP^ structure (gray; PDB: 4DST) indicating the key structural motifs and mapping of residues undergoing significant CSP onto the ribbon and surface representation of KRAS G12D, as in A, in pink and magenta. Residues with significant and the highest CSP concentrate into a groove (dotted circle line) that is formed in the switch-I/II helix region and termed allosteric site. **C,** Close-up view of the allosteric binding site and bound docked structure of KRB-456 (yellow). Potential hydrogen bonds and van der Waals contacts between KRB-456 and KRAS G12D residues are illustrated. **D,** RMSD analysis during the MD simulation of the KRAS G12D–KRB-456 complex shown in C, indicating small and low amplitude RMSD values for KRB-456. **E,** KRAS G12D–KRB-456 interaction maps from 20, 40, 60, and 80 ns snapshots during the MD simulation showing a multitude of direct and water-mediated hydrogen bonds and van der Waals contacts between KRB-456 and allosteric pocket residues.

To better understand the binding interaction of KRB-456 with KRAS G12D, we performed two-dimensional (2D) ^1^H-^15^N HSQC NMR analysis of ^15^N-labeled KRAS G12D bound to GDP and the non-hydrolysable nucleotide analog of GTP, GCP (β,γ-Methyleneguanosine 5′-triphosphate). HSQC-NMR experiments showed that KRAS G12D adopts a folded conformation (Fig 4A) [residue assignments were transferred from similar previously published (BMRB ID 27719) data of KRAS G12D]. Titration of the KRB-456 compound to KRAS G12D^GDP^ conformation demonstrated several chemical shift perturbations (CSPs) in different regions of the protein including switch-II and I and their adjacent regions suggesting direct binding effects but also indirect effects by KRB-456 binding (Fig. 4A). The highest CSPs in surface exposed residues were predominantly localized in the switch-II region and in adjacent residues of the central β-sheet that comprise a binding pocket (Fig. 4A and B). Furthermore, titration of the KRB-456 compound to KRAS G12D^GCP^ conformation showed CSPs around the switch-II region, as in KRAS G12D^GDP^ conformation, but CSPs were weaker and showed distinct dynamic effects in the switch-I and P-loop regions of KRB-456 in KRAS G12D^GCP^ conformation (Supplementary Fig. S2A and S2B).

To support the identification of the pocket for KRB-456 binding, the crystal structures of KRAS G12D^GDP^ and G12D^GCP^ conformations were compared. Residues K5, L56, Y71, and T74 are part of a pocket formed within the KRAS G12D^GCP^ conformation; however, these residues do not form an obvious pocket within the KRAS G12D^GDP^ conformation (Supplementary Fig. S3A). Interestingly, molecular dynamics (MD) simulations of the KRAS G12D^GDP^ conformation over 100 ns showed the same residues can form a well-defined pocket on the surface of KRAS G12D^GDP^. Moreover, analysis of this pocket from a KRAS G12D^GDP^ 100 ns snapshot and KRAS G12D^GCP^ crystal structure indicated similar pocket volumes, suggesting that this pocket is dynamic and can be present either with the GDP- or GCP-bound conformation of KRAS G12D (Supplementary Fig. S3A). The plasticity of this allosteric pocket is likely due to the conformational flexibility of Y71 sidechain on switch-II helix that can move away from the central β-sheet and allow formation of the pocket with either GDP or GCP bound to KRAS G12D (Supplementary Fig. S3B and S3C).

We next performed molecular docking of KRB-456 around the allosteric pocket and in nearby residues suggested by the NMR studies to account for potential ambiguity in the NMR data. Using an induced-fit docking approach (44), KRB-456 was docked preferentially to the switch-I/II allosteric pocket, which is the same pocket suggested by binding site analysis (Fig. 4C; Supplementary Fig. S2A). KRB-456 forms several van der Waals contacts with its hydrophobic side and the base of the switch-I/II allosteric pocket but also potential hydrogen bonds with polar sidechains of residues such as S39 and K5 (Fig. 4C). Consistent with this binding mode, E37, Y40, D54, L56, Y71, T74, G75, and E76 undergo significant CSP upon titration of KRB-456 (Fig. 4A). To further assess the docked position and dynamics of KRB-456 within the pocket, we performed MD simulations. In MD simulations, KRB-456 binding mode presented dynamic fluctuations within the allosteric pocket but kept the interactions with the residues of the pocket and switch hydrogen bonding interactions between pocket residues and water molecules (Fig. 4D and E).

To further confirm the specificity of the KRB-456 for binding to the switch-I/II allosteric binding, we performed HSQC-NMR analysis of KRAS G12D^GDP^ upon IIA-15D titration. Consistent with the ITC data that showed reduced affinity for IIA-15D compared with KRB-456, we also observed reduced CSPs for IIA-15D compared with KRB-456 (Supplementary Fig. S4A). Structural comparison of IIA-15D with the KRB-456 docked pose to KRAS G12D^GDP^ suggested that although the IIA-15D can form similar interactions with the base of the switch-I/II allosteric pocket, its bulky phenyl ring, instead of the methyl ester of KRB-456, introduces steric clash with the pocket residues resulting in reduced affinity (Supplementary Fig. S4B).

We have also compared the available KRAS G12C structures with KRAS G12D structures either bound to GDP or GCP. The overall fold of the structures and the majority of the secondary structure segments overlay significantly with the exception of switch-II loop, switch-II helix, and switch-I region where there is more conformational variability. This is consistent with the flexibility of the switch I and switch II regions that undergo conformational fluctuations upon mutations or ligand binding. Therefore, it is likely that G12C mutant structure generates some constraint on the structure that does not enable productive binding or induce fit of the KRB-456 to the switch-I/II allosteric pocket while this is accomplished with the G12D mutant structure.

### BI-2852 Competes with KRB-456 for Binding to KRAS G12D

The above protein NMR studies revealed that KRB-456 binds KRAS G12D by forming interactions with a dynamic allosteric binding pocket within the switch-I/II region. To provide further evidence for this, we performed a binding competition experiment with BI-2852, a chemical probe that was shown to bind to a pocket between switch I and II and that has a Kd of 740 nmol/L (ITC) for KRAS G12D. To this end, we preincubated for 15 minutes KRAS G12D with increasing concentrations of BI-2852 prior to determining the Kd of KRB-456 binding to KRAS G12D using ITC as described in Materials and Methods. In the absence of preincubation with BI-2852 (preincubation with vehicle control only), KRB-456 bound KRAS G12D with a Kd of 285 nmol/L (Supplementary Fig. S5A). Preincubation with 500, 750, and 1,000 nmol/L of BI-2852 decreased the affinity of KRB-456 for KRAS G12D as demonstrated by a concentration-dependent increase in its Kd to 426, 692, and 2850 nmol/L, respectively (Supplementary Fig. S5A and S5B). Furthermore, the binding stoichiometry between KRB-456 and the KRAS G12D protein decreased by 10-fold from about “1:1” to about “0.1:1” (Supplementary Fig. S5B). These data suggest that KRB-456 binds to a pocket that is no longer accessible when BI-2852 is bound to KRAS G12D, consistent with our protein NMR and molecular docking results. Indeed, comparison of the KRAS G12D GCP co-crystal structure bound to BI-2852 (25) with the KRB-456–bound structural model of KRAS G12D GCP showed significant overlap of the two KRAS structures and both BI-2852 and KRB-456 compounds interacting with the allosteric site (Supplementary Fig. S5C).

### KRB-456 Suppresses the Levels of KRAS Bound to GTP, Inhibits the Binding of KRAS to RAF1, and Deceases P-MEK and P-ERK Levels in Human Pancreatic Cancer Cells

**FIGURE 5:**
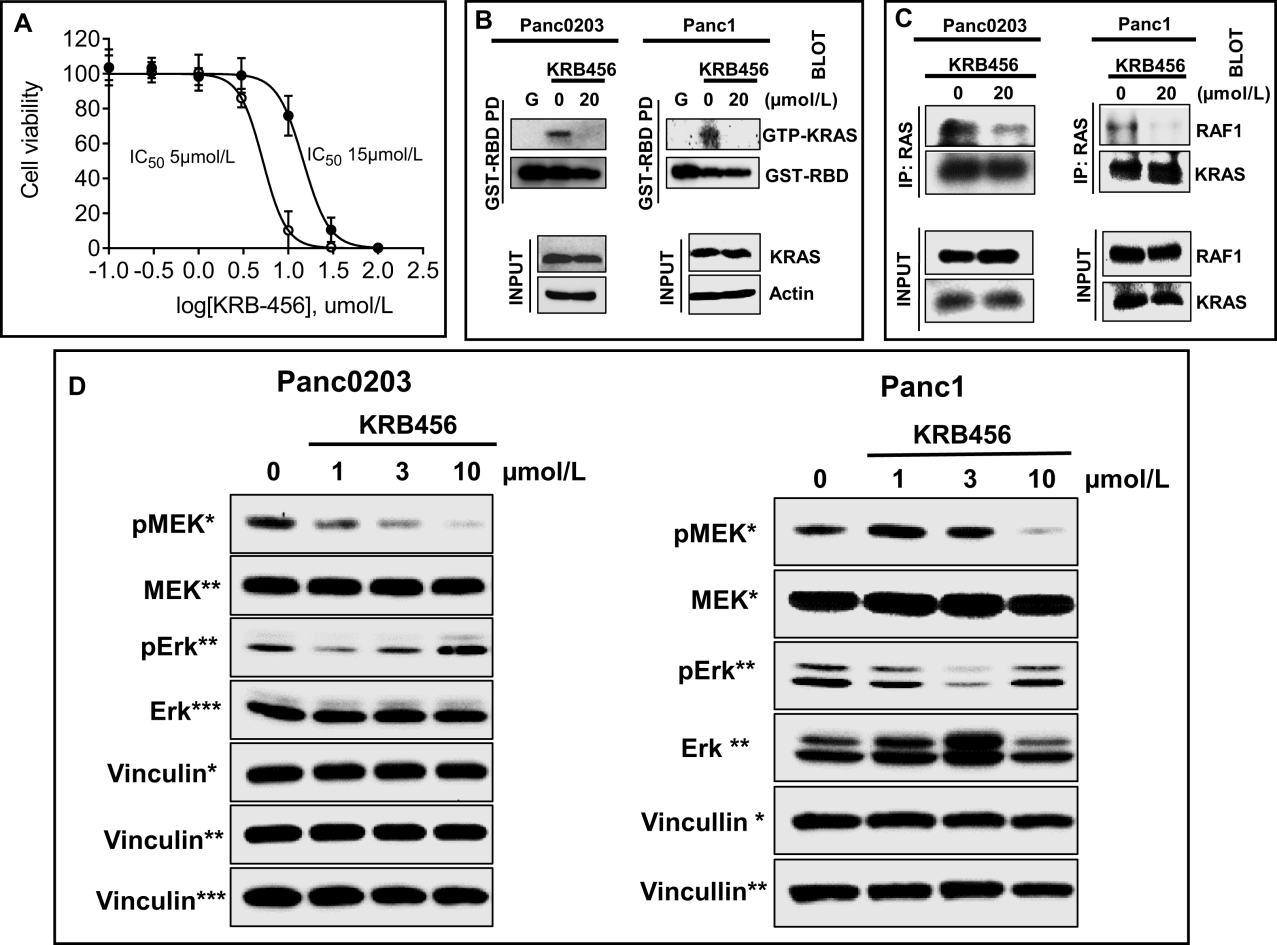
Effects of KRB-456 on cell viability, levels of KRAS bound to GTP, binding of KRAS to RAF1, and the levels of P-MEK and P-ERK in human pancreatic cancer cells that harbor KRAS G12D. Human pancreatic cancer cell lines Panc0203 and Panc1 were treated with the indicated concentrations of KRB-456 and processed for viability assays (72 hours), Panc0203 (open circles) and Panc1 (closed circles; **A**), GST-RBD pulldown (“G” designates GST-RBD only with no lysate added; **B**), immuno-precipitation with RAS antibody and blotting with RAF1 and KRAS antibodies (**C**), and Panc0203 and Panc01 cells were treated with 0, 1, 3, and 10 µmol/L KRB-456 for 2 hours and processed for Western blotting as described in Materials and Methods (**D**).

The results shown in Figs. 1–4 and Supplementary Figs. S2–S5 demonstrate that KRB-456 binds to KRAS G12D and blocks its binding to GST-RBD. We next examined the effects of KRB-456 on the levels of KRAS bound to GTP and on the binding of KRAS to RAF1 in intact Panc0203 and Panc1 human pancreatic cancer cells that harbor mt KRAS G12D. To this end, we first treated these cells with various concentrations of KRB-456 and processed the cells for viability assays, immunoprecipitation, GST-RBD pulldown, and western blotting as described in Materials and Methods. Figure 5A shows that KRB-456 inhibited the viability of Panc0203 and Panc1 cells in a concentration-dependent manner with IC_50_ values of 4.9 ± 0.7 µmol/L and 14.8 ± 2.7 µmol/L, respectively. Similar IC_50_ values were obtained with other pancreatic cancer cell lines including one that harbors KRAS G12D (Panc0403 cells, IC_50_ = 12.61 µmol/L), KRAS G12V (Capan1 cells, IC_50_ = 10.46 µmol/L), KRAS G12C (MiaPaCa2, IC_50_ = 12.68 µmol/L), and KRAS WT (BxPc3 cells, IC_50_ = 11.83 µmol/L; Supplementary Fig. S6).

We next treated Panc0203 and Panc1 cells with KRB-456 for 15 minutes, lysed the cells, and determined the effects of KRB-456 on the levels of GTP-bound KRAS by GST-RBD pulldown assays and the binding of KRAS to RAF1 by co-immunoprecipitation assays as described in the Materials and Methods. Figure 5B shows that GST-RBD was able to pull down GTP-bound KRAS in Panc0203 and Panc1 cells treated with vehicle, and that treatment with KRB-456 significantly decreased the GTP-KRAS cellular levels. KRB-456 also inhibited the binding of KRAS to RAF1 in intact Panc0203 and Panc1 cells (Fig. 5C) confirming the *in vitro* AlphaScreen and GST pulldown results of Figs. 1 and 2. Next, we determined the effects of KRB-456 on the phosphorylation levels of the immediate downstream effector of RAF1, MEK, as well as the phosphorylation levels of ERK. To this end, we treated Panc0203 and Panc1 cells with various concentrations of KRB-456 (0, 1, 3, and 10 µmol/L) and processed the cells for Western blotting as described in Methods. Figure 5D shows that treatment of Panc0203 cells with KRB-456 significantly decreased P-MEK levels in a concentration-dependent manner starting at 1 µmol/L. In Panc1 cells, KRB-456 increased P-MEK slightly at 1 and 3 µmol/L by 1.4- and 1.1-fold, respectively, before significantly decreasing these levels at 10 µmol/L (Fig. 5D). In Panc0203 and Panc1 cells, KRB-456 decreased P-ERK levels at the lower doses but not at the higher dose of 10 µmol/L (see Discussion section). We also treated Panc0203 and Panc1 cells with KRB-456 for various periods of time and processed the cells for Western blotting as described in Materials and Methods. Supplementary Figure S7A and S7B show that treatment of Panc0203 and Panc1 cells with KRB-456 transiently increased the P-MEK levels within 5 min, which peaked at 15 minutes followed by a steady decrease starting at 30 minutes, with significant decreases of the P-MEK levels between 8 and 48 hours. Similar results were observed with P-ERK levels rapidly and transiently increasing followed by significant decreases in these levels within 30 minutes of KRB-456 treatments of Panc0203 and Panc1 cells.

### KRB-456 Inhibits the Growth *In Vivo* of Subcutaneous and Orthotopic mt KRAS Tumors Derived From Patients with Pancreatic Cancer

**FIGURE 6:**
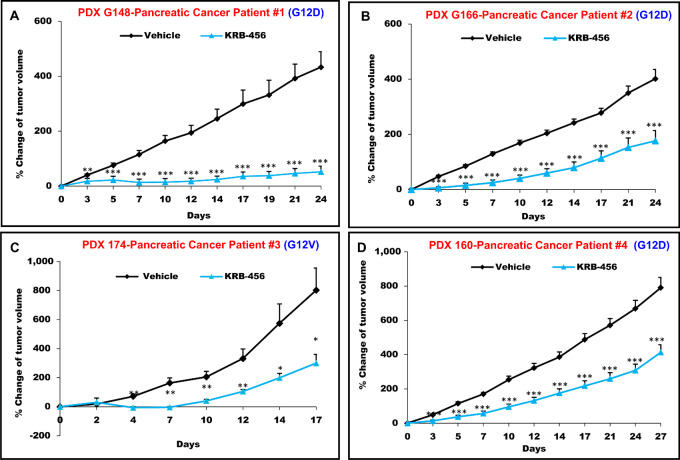
KRB-456 inhibits the growth of mt KRAS xenografts from patients with refractory pancreatic cancer. Fresh tumor biopsies from 4 patients with pancreatic cancer were prepared as described in Materials and Methods and implanted subcutaneously into NSG mice, and when the average tumor volumes were about 200 mm^3^, the mice were randomized and treated daily with vehicle (V) or KRB-456 5 mpk daily for 24 days, except for days 12, 13 with no treatment (**A**); 5 mpk daily for 24 days except for days 8, 10 and 19, 21 with no treatment (**B**); 5 mpk daily for 17 days except for days 11, 12 and 14 to 17 with no treatment (**C**), and 4 mpk with intermittent cycles of 5 days of treatment followed by 2 days of no treatment for a total of 27 days (**D**). (*, *P* < 0.05; **, *P* < 0.01; ***, *P* < 0.001). *P* values determined by Student *t* test. Error bars represent SE. The 4 and 5 mpk doses were chosen based on our results of MTD studies that are described in Materials and Methods. The % change of tumor volume for each tumor was calculated as: {[(Volume at day X) − (Volume at day 0)]/(Volume at day 0)} × 100, with X being any day of tumor measurement. The average tumor volumes at the start of the experiment in Fig. 6 are: Patient 1 PDX: Vehicle (176 ± 59) and KRB456 (175 ± 65 mm^3^); Patient 2 PDX: Vehicle (204 ± 139) and KRB456 (202 ± 139 mm^3^); Patient 3 PDX: Vehicle (299 ± 156) and KRB456 (309 ± 152 mm^3^); Patient 4 PDX: Vehicle (183 ± 76) and KRB456 (182 ± 81 mm^3^).

We next determined whether KRB-456 can inhibit the growth in mice of PDXs. To this end, we used fresh biopsies from 4 patients with pancreatic cancer, 3 patients (G166, G148, and G160) whose tumors harbor KRAS G12D mutations, and 1 patient (G174) whose tumor harbors KRAS G12V mutation (see Materials and Methods section for patients age, sex, tumor pathology, staging, and patient treatments). To determine the effects of KRB-456 on the growth *in vivo* of subcutaneously implanted tumors, we implanted subcutaneously freshly resected tumor biopsy from all 4 patients in NSG mice, and randomized into vehicle groups and KRB-456 groups for each of the patient PDXs, and treated the mice as described by us (39) and in the Materials and Methods. By day 24 of the vehicle-treated mice, Patient G148 PDX (Fig. 6A) and Patient G166 PDX (Fig. 6B) showed average growth of 401% and 433%, respectively. The vehicle-treated mice group of Patient G174 PDX (Fig. 6C) grew much faster with average growth of 802% by only 17 days, and the experiment was stopped by day 17 due to animal protocol tumor size limits. The vehicle-treated group of Patient G160 PDX (Fig. 6D) had an average growth of 791% but not until day 27. In all KRB-456–treated mice, tumor growth was statistically significantly inhibited as early as day 3 of treatment and the average growth was only 52%, 177%, 300%, and 413%, by the end of the KRB-456 drug treatments as compared with 401%, 433%, 802%, and 791%, respectively, in the corresponding vehicle-treated groups (Fig. 6). Therefore, KRB-456 inhibited tumor growth by 7.7-, 2.5-, 2.7-, and 1.9-fold, respectively, demonstrating that KRB-456 is effective at inhibiting the growth of pancreatic cancer patient PDXs implanted subcutaneously.

To determine whether KRB-456 can also inhibit orthotopic tumor growth, tumor biopsies from patient G160 were implanted orthotopically in NSG mice as described in Materials and Methods and as described by us previously (39). G160 implanted orthotopically grew much slower than when implanted subcutaneously (compare Fig. 6D with Supplementary Fig. S8A). On day 20 after orthotopic tumor implantation, mice were treated intraperitoneally daily for 30 days with vehicle or 5 mpk KRB-456, after which the mice were monitored for an additional 47 days without treatment. Tumors were monitored by ultrasound, as described in Materials and Methods, starting on day 40, but only became large enough to be measurable starting on day 54, and tumor measurements continued until day 97 after implantation. Supplementary Figure S8A shows average tumor volumes of the vehicle and the KRB-456 groups from day 54 to day 97, and Supplementary Fig. S8B shows representative ultrasound tumor images on days 54 and 97 from both vehicle- and KRB-456-treated mice. Both Supplementary Fig. S8A and S8B show that treatment with KRB-456 inhibited the growth of the orthotopically-implanted PDX from patient G160. Specifically, Supplementary Fig. S8A shows that by day 97, the tumors from the vehicle-treated mice grew to an average tumor volume of 504.7 ± 106.42 mm^3^. In contrast, the tumors from the KRB-456–treated mice grew to an average tumor volume of only 151.95 ± 23.27 mm^3^ (Supplementary Fig. S8A). Therefore, KRB-456 inhibited tumor growth by 70%, demonstrating that KRB-456 is effective at inhibiting not only subcutaneous PDX tumor growth (Fig. 6A–D) but also the orthotopic growth of the tumor derived from patients with pancreatic cancer.

### KRB-456 Inhibits P-MEK, P-AKT, and P-S6 Levels and Induces Apoptosis *In Vivo* in KRAS G12D PDXs from Patients with Pancreatic Cancer

**FIGURE 7:**
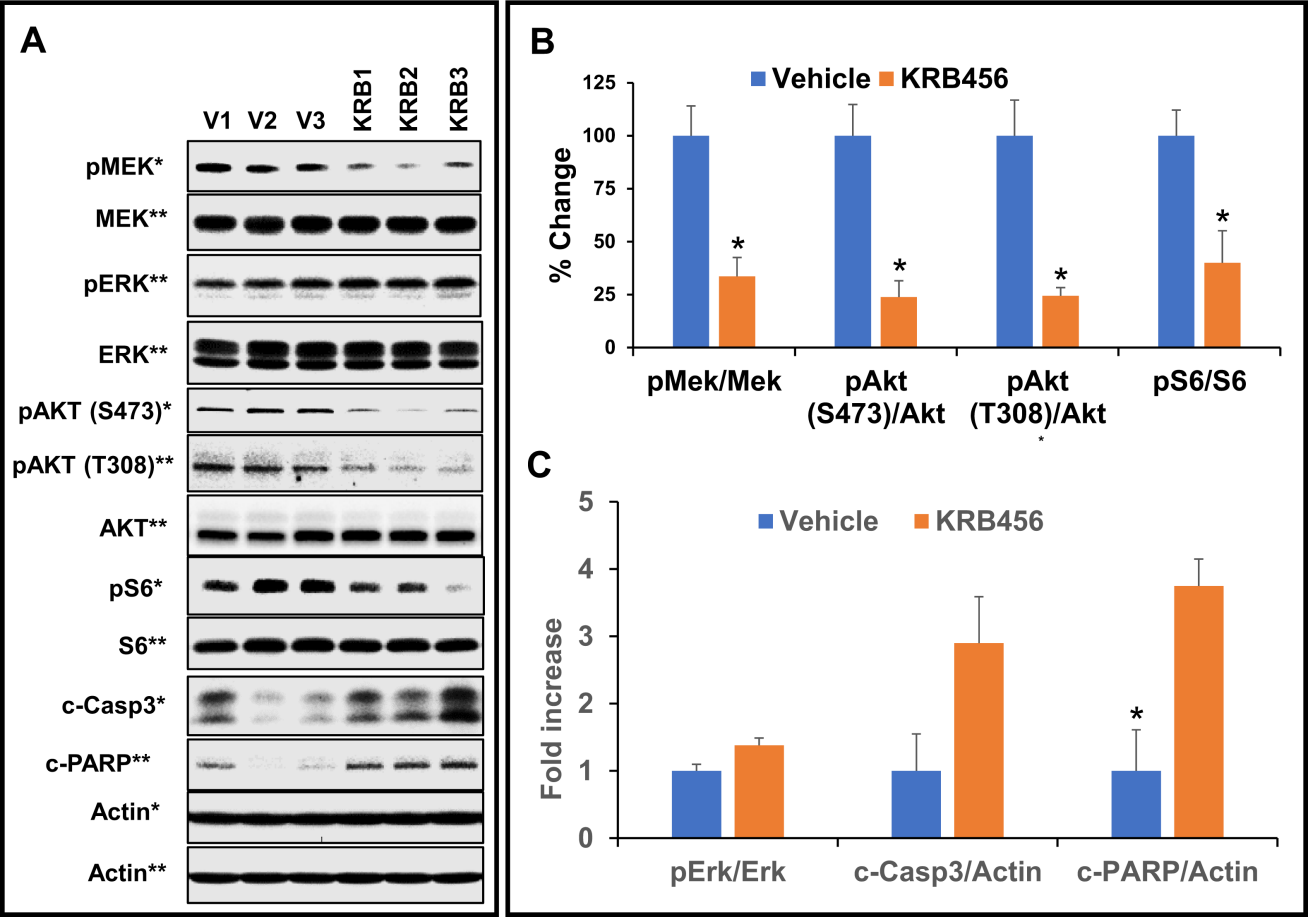
Treatment of mice with KRB-456 inhibits P-MEK, P-AKT, and P-S6 levels and induces apoptosis in KRAS G12D PDXs from patients with pancreatic cancer. **A,** Tumor biopsies from patient G160 were subcutaneously implanted into NSG mice as described in Materials and Methods, and when the average tumor volume reached 200 mm^3^, the mice were treated either with vehicle (mice V1, V2, V3) or 5 mpk KRB-456 (mice KRB1, KRB2, KRB3) as described in Materials and Methods. Two hours after treatment, tumors were harvested and lysates were processed for Western blotting as described in Materials and Methods. * and ** designates blots from different gels. **B** and **C,** Quantification of the Western blots. *P* values were determined by Student *t* test, * designates *P* < 0.05. The actual *P* values were 0.016 (P-MEK/MEK), 0.010 (P-ATK(S473)/AKT), 0.012 (P-ATK(T308)/AKT), 0.037 (P-S6/S6), 0.069 (P-ERK/ERK), 0.097 (CASPASE3/b-ACTIN), and 0.019 (PARP/b-ACTIN). Error bars represent SE.

To determine whether KRB-456 inhibits signal transduction pathways downstream of RAS following mice treatments, tumor biopsies from patient G160 were implanted subcutaneously in NSG mice, and when the average tumor volume reached 200 mm^3^, the mice were randomized and treated either with vehicle (3 mice, V1, V2, and V3) or 5 mpk KRB-456 (3 mice, KRB1, KRB2, and KRB3) as described in Materials and Methods. Two hours after treatment, tumors were harvested and lysates were processed for Western blotting as described in Materials and Methods. As shown in Fig. 7A, KRB-456 mouse treatment decreased the levels of P-MEK, P-AKT, and P-S6 but not P-ERK (see Discussion) in the pancreatic cancer PDX G160 *in vivo*. Quantification of these results shows that KRB-456 significantly inhibited the levels of P-MEK, P-AKT (S473), P-AKT (T308), and P-S6 by 66.4%, 76.2%, 75.6%, and 60.0%, respectively (Fig. 7B). In addition, KRB-456 also increased the levels of cleaved caspase 3 and PARP by 2.9-fold and 3.8-fold, respectively. Similar results were also obtained with a PDX G166 from another patient. Supplementary Figure S9 shows that KRB-456 inhibited the levels of P-MEK, P-AKT (S473), P-AKT (T308), and P-S6 by 49.9%, 73.5%, 56.7%, and 83.9%, respectively, and increased the levels of cleaved caspase 3 and PARP by 3.1-fold and 2.7-fold, respectively. These data suggest that inhibition of P-MEK, P-AKT, and P-S6 resulted in apoptosis induction in these pancreatic cancer PDXs, which in turn could have contributed to the observed tumor growth inhibition.

### KRB-456 Inhibits the Viability in 3D Cocultures of Primary and Metastatic mt KRAS Adenocarcinoma Cells Derived From 8 Patients with Pancreatic Cancer

We next determined the efficacy of KRB-456 to inhibit the viability, under standard and 3D coculture models, of low-passage pancreatic cancer cells that were generated from patients with pancreatic cancer as described by us previously (41, 42). These cell lines were derived from primary and metastatic human tumors from 8 patients with pancreatic cancer (patient nos. 43, 53, 66, 69, 102, 107, 108, and 124; ref. 43). The efficacy of KRB-456 to inhibit the viability of the eight patient-derived tumor cell lines was first determined in 2D standard conditions as described under Materials and Methods. Supplementary Figure S10A shows representative live-cell images of cells derived from patient 107 where KRB-456 inhibited the viability of these pancreatic cancer cells in a dose-dependent manner. Supplementary Figure S10B shows that KRB-456 inhibited the viability under 2D conditions of cells derived from all 8 patients with IC_50_ values ranging from 4.7 to 16.7 µmol/L. Importantly, KRB-456 was just as effective when the patient-derived tumor cells were cultured in 3D conditions alone or when cocultured with the chemoresistance-promoting human pancreatic stellate cells harvested from pancreatic adenocarcinoma cells with IC_50_ values ranging from 3.8 to 18.2 µmol/L (Supplementary Fig. S10B).

## Discussion

Recently, major advances have been made toward the development of drugs that target tumors that harbor mt KRAS G12C with two inhibitors, sotorasib and adagrasib, FDA approved for patients with advanced NSCLC harboring KRAS G12C mutation (14–17, 22, 23, 45). However, there are no inhibitors that have been FDA approved for the more common KRAS G12D and KRAS G12V. In this study, using AlphaScreen, GST-RBD pulldown, ITC and protein NMR, we discovered a novel small molecule, KRB-456, that binds a switch-I/II allosteric pocket of KRAS G12D. KRB-456 binds with high affinity to KRAS G12D and KRAS G12V, decreases the cellular levels of GTP-bound KRAS, inhibits the binding of KRAS to RAF1 in pancreatic cancer cells, and inhibits *in vivo* tumor growth of subcutaneous and orthotopic xenografts from patients with pancreatic cancer. Furthermore, KRB-456 could also potentially be efficacious against pancreatic cancer tumors harboring other mutations. Indeed, although in ITC *in vitro* assays using purified KRAS proteins, KRB-456 binds to KRAS G12D with 1.5-, 2-, and 6-fold higher affinity than to KRAS G12V, KRAS WT, and KRAS G12C, respectively, its potency to inhibit cell viability of pancreatic cancer cell lines harboring KRAS G12D, KRAS G12V, KRAS WT, and KRAS G12C were similar with IC_50_ values ranging from 5 to 15 µmol/L (see Results section). The similar potency to inhibit the viability of KRAS G12D- and KRAS G12C-harboring cancer cell lines despite the higher affinity of binding by ITC assays to KRAS G12D over KRAS G12C could be due to KRB-456 inhibiting cell viability by not only binding, in the same cell line, to the mutant KRAS allele but also to the WT KRAS allele, which in ITC assays has a similar affinity to KRAS G12D.

KRB-456 was identified from a natural product-inspired library of structurally diverse small molecules with high structural and stereochemical complexity (30). Out of the six confirmed hits from the 560-compound library, the four most potent were derivatives of the same pharmacophore, which allowed SAR results that indicated that halide methyl groups in the R2 position are preferred to acetate groups. Furthermore, the smaller methyl ester group in the R1 position is preferred to the bulkier phenyl group, possibly due to steric hindrance, which was corroborated by our protein NMR studies.

ITC and NMR studies demonstrated that KRB-456 binds both the GDP-bound and GCP-bound form of KRAS G12D. Our NMR and MD studies showed that KRB-456 binds an allosteric pocket formed mainly by switch-I/II residues, and that this pocket has flexibility to undergo dynamic fluctuations between a closed and open conformation. These studies also showed that KRB-456 is capable of binding and inducing an open conformation, which is typically observed in active GTP-bound conformations of KRAS crystal structures. This is consistent with the functional activity of KRB-456 to inhibit effector RAF1 binding. NMR studies also show broader indirect effects around switch-I and -II regions that allosterically can induce RAF1 binding inhibition. Furthermore, structural studies also support that RAF RBD binding to KRAS can overlap with the boundaries of this allosteric pocket (46) and therefore KRB-456 may directly inhibit RAF1 binding. Other studies have also shown fragments and small molecules to engage similar residues between the switch-I and -II region and block effector binding as well as binding of GEF and GAP proteins to RAS (25, 47, 48). To provide further evidence for binding of KRB-456 to this allosteric pocket within the switch-I/II region, we demonstrated that BI-2852, a chemical probe that was shown to bind to a pocket between switch-I and -II regions (25), competes for binding of KRB-456 to KRAS G12D. Furthermore, comparison of the KRAS G12D GCP co-crystal structure bound to BI-2852 (25) with the KRB-456–bound structural model of KRAS G12D GCP showed significant overlap of the two KRAS structures and indicated that both BI-2852 and KRB-456 interact with the same allosteric site. An intriguing feature of KRB-456 is that, despite its small size, it possesses high structural diversity represented by the hydrocarbon core and polar groups that enable the formation of several van der Waals and hydrogen bond contacts, respectively, which also matches with the properties of the switch-I/II allosteric pocket. Nevertheless, the plasticity of this allosteric site suggests that further optimization of the KRB-456 interactions can be achieved. Furthermore, solving the KRB-456–KRAS complex structure using X-ray crystallography could facilitate the design of KRB-456 derivatives that are more potent and selective possibly leading to clinical candidates.

The recent FDA approval of drugs that selectively target KRAS G12C is a significant accomplishment that has a major impact on patients with cancer whose tumors harbor KRAS G12C mutations. However, discovering drugs that could benefit patients with cancer whose tumors harbor KRAS G12D and KRAS G12V mutations remains a significant unmet need, because the prevalence in human cancers of these mutations is higher than that of KRAS G12C mutations (9). Therefore, discovering an inhibitor, such as KRB-456, which binds KRAS G12D and KRAS G12V, will have a broader impact on human cancers, particularly in PDAC, colorectal cancer, and other cancers where the incidence of KRAS G12D and KRAS G12V mutations is significant. Our studies show that KRB-456 at a low dose (5 mpk) was effective at inhibiting the tumor growth of PDXs from pancreatic cancer biopsy samples implanted subcutaneously and orthotopically in mice. Importantly, KRB-456 inhibited the *in vivo* growth of PDXs from patients with pancreatic cancer with disease recurrence after chemotherapy and radiotherapy. Furthermore, KRB-456 was also effective at inhibiting the viability of primary and metastatic mt KRAS tumor cells (derived from 8 patients with pancreatic cancer) in 3D cocultures with pancreatic stellate cells, precursor cells of cancer-associated fibroblasts that reside in the tumor microenvironment, and that promote tumor proliferation and metastasis as well as drug resistance. These 3D coculture results, coupled with the *in vivo* PDX results showing that KRB-456 is effective against tumors derived from relapsed patients (two of the biopsies were resected from patients who relapsed after treatment with 5-FU, gemcitabine, capecitabine, and/or radiation), suggest that KRB-456 can overcome resistance to chemotherapy and radiotherapy.

KRB-456 binds KRAS (ITC and protein NMR data), inhibits the binding of KRAS to RAF1 *in vitro* (alpha screen and GST-RBD pulldown) as well as in intact cancer cells (co-immunoprecipitation with KRAS and blotting with RAF-1 and GST-RBD pulldown) and inhibits the immediate RAS/RAF downstream effector P-MEK clearly demonstrating that KRB-456 engages its target and suppresses KRAS oncogenic signaling in pancreatic cancer. This, coupled with the demonstration that treatment of mice bearing mutant KRAS pancreatic PDXs with KRB-456 inhibits P-MEK and P-AKT and induces apoptosis at doses that are tolerable, suggests that KRB-456 inhibits tumor growth at least in part by inhibiting KRAS oncogenic signaling. Furthermore, KRB-456 significantly decreased the levels of P-S6 and this may also contribute in part to the ability of KRB-456 to inhibit tumor growth of these PDXs. This is consistent with studies showing that inhibition of the phosphorylation levels of S6 is closely associated with inhibition of tumor growth *in vivo* and that sustained activation of mTORC1, as measured by P-S6 levels, was shown to contribute greatly to resistance to targeted therapies (49).

Time course studies in cultured cells showed that KRB-456 treatment of Panc0203 and Panc1 cells causes rapid (5–15 minutes) and transient increases in the P-MEK and P-ERK levels followed within 30 minutes by a steady and significant decrease of these levels. The mechanism by which this occurs is not known. Furthermore, dose concentration response studies showed that at higher concentrations, KRB-456 treatment of Panc1 and Panc0203 cells decreased the levels of P-MEK, but not P-ERK. Importantly, *in vivo* studies demonstrated that treatment with KRB-456 of mice bearing PDXs from pancreatic cancer patients resulted in inhibition of the PDX's P-MEK but not P-ERK levels. The reason for this lack of inhibition of P-ERK levels is not known. One possible explanation is that KRB-456 could induce an adaptive reactivation of P-ERK as was shown previously with KRAS G12C inhibitors (22, 23, 50). While the mechanism of the lack of inhibition of P-ERK is yet to be uncovered, our studies suggest that the combination of KRB-456 with ERK inhibitors may prove to be more efficacious than single-agent treatment. Furthermore, three reports (51–53) showed that in KRAS-driven cancers such as pancreatic cancer, RAF/MEK/ERK kinase cascade and the autophagy pathways cooperate to maintain tumor survival and, as such, the combined inhibition of both pathways was highly efficacious at inhibiting tumor growth. Therefore, at least in the case of pancreatic cancer where the tumors appear to be highly dependent on energy from autophagy especially when KRAS or its downstream effectors are inhibited, combination of KRB-456 with autophagy inhibitors may also prove to be efficacious.

In summary, we report here on the discovery of a novel natural product-inspired small molecule, KRB-456, that binds a dynamic allosteric binding pocket within the switch-I/II region of KRAS G12D, suppresses the levels of KRAS bound to GTP and inhibits the binding of KRAS to RAF1 in human pancreatic cancer cells that harbor KRAS G12D. The fact that KRB-456 thwarted the subcutaneous and orthotopic growth *in vivo* of PDXs from patients with pancreatic cancer that relapsed after chemotherapy and radiotherapy suggests that it may overcome drug resistance of these tumors.

### Statement of Translational Relevance

At present, there are no clinically approved drugs directly thwarting mutant KRAS G12D, a major driver of oncogenesis and therapy resistance. Here, we report on the discovery of a novel small molecule, KRB-456, that binds a dynamic allosteric binding pocket within the switch-I/II region of KRAS G12D. KRB-456 binds KRAS G12D with 1.5-, 2-, and 6-fold higher affinity than KRAS G12V, KRAS WT, and KRAS G12C, respectively. KRB-456 inhibits KRAS G12D binding to RAF1 and KRAS signaling in cultured human pancreatic cancer cells. In mouse models, KRB-456 inhibits P-MEK, P-AKT, and P-S6 levels *in vivo* and inhibits the growth of subcutaneous and orthotopic xenografts derived from patients with pancreatic cancer whose tumors harbor KRAS G12D and KRAS G12V and who relapsed after chemotherapy and radiotherapy. This discovery opens new avenues to target KRAS G12D- and KRAS G12V-driven pancreatic cancer, and as such it warrants further advanced preclinical and clinical studies.

## Supplementary Material

Figure S1 KRB-456 analogs IB-21G, IB-21J and IIA-15D binds KRAS G12D with lower affinity.

Figure S2 KRB-456 binds to KRAS G12DGCP at a switch-I/II allosteric site.

Figure S3 KRAS switch-I/II allosteric pocket is dynamic.

Figure S4 IIA-15D shows reduced chemical shift perturbations to KRAS G12DGDP possibly due to steric clash with the KRAS allosteric pocket.

Figure S5 BI-2852 competes with KRB-456 for binding to KRAS G12D.

Figure S6 Effects of KRB-456 on cell viability in human pancreatic cancer cells that harbor KRAS G12D (Panc0403 cells), KRAS G12V (Capan1 cells), KRAS G12C (MiaPaCa2 cells), and KRAS WT (BxPc3 cells).

Figure S7 Effects of KRB-456 on P-MEK and P-ERK levels in human pancreatic cancer Panc0203 and Panc1 cells.

Figure S8 KRB-456 inhibits the growth in vivo of orthotopic mt KRAS tumors derived from pancreatic cancer patients.

Figure S9 Treatment of mice with KRB-456 inhibits P-MEK, P-AKT and P-S6 levels and induces apoptosis in KRAS G12D PDXs from pancreatic cancer patients.

Figure S10 KRB-456 inhibits the viability in 2D, 3D, and 3D co-cultures with pancreatic stellate cells (PSCs), of primary and metastatic mt KRAS adenocarcinoma cells derived from 8 pancreatic cancer patients.

Supplementary Figure Legends Supplemental Figure Legends

Supplementary Methods Supplemental Methods

Supplementary Table S1 Structure-activity relationship studies of the inhibition of KRAS G12D/GST-RBD binding.
