# Supplementary material for: Discovery of KRB-456, a KRAS G12D Switch-I/II Allosteric Pocket Binder That Inhibits the Growth of Pancreatic Cancer Patient-derived Tumors
Source: Cancer Res Commun. 2023 Dec 28;3(12):2623–39. doi: 10.1158/2767-9764.CRC-23-0222 (PMC10754035; doi:10.1158/2767-9764.CRC-23-0222)
Supplement: Figure S1 — KRB-456 analogs IB-21G, IB-21J and IIA-15D binds KRAS G12D with lower affinity. [file crc-23-0222-s01.pptx]

## Slide 1
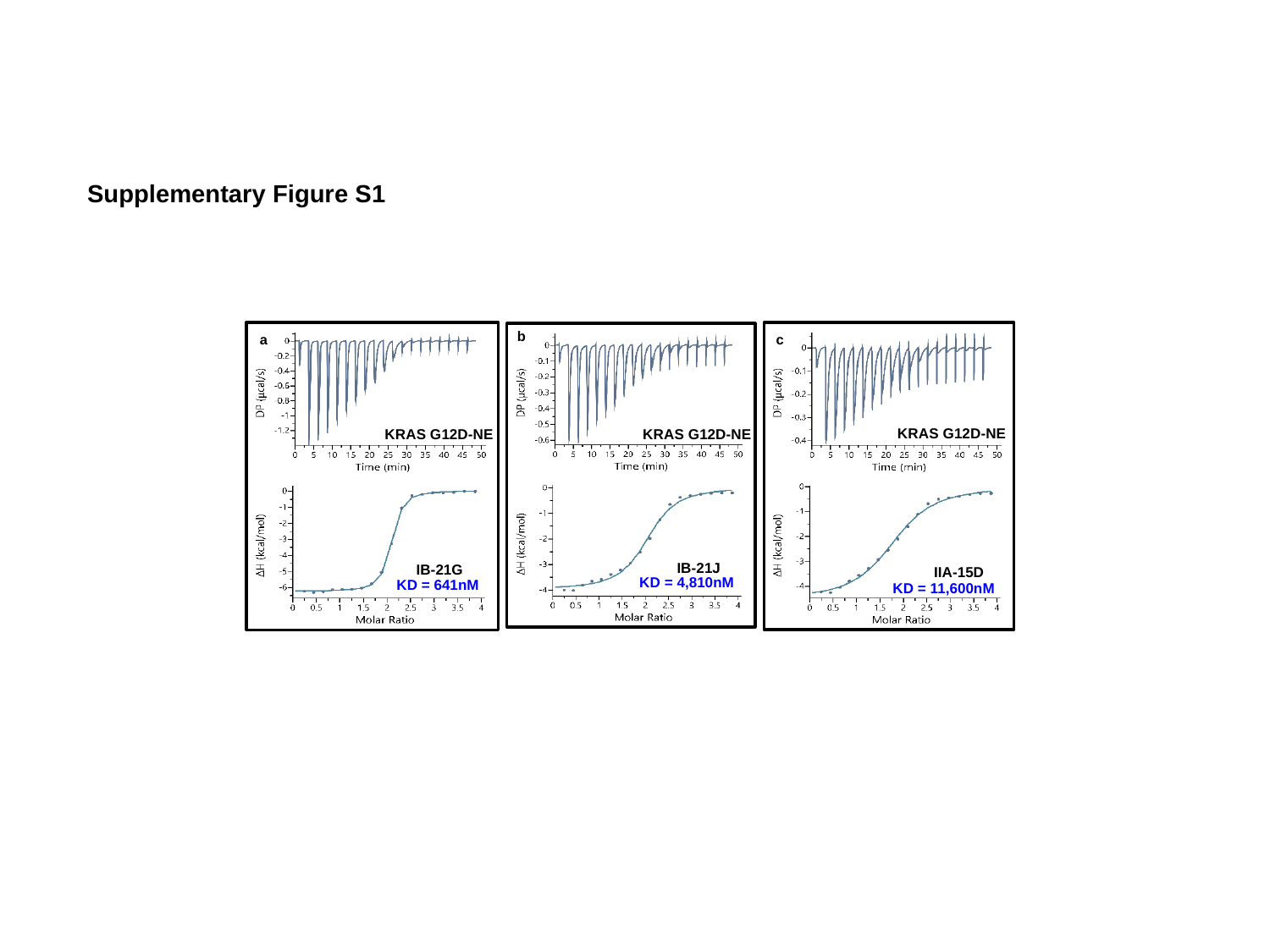

CONFIDENTIAL
Figure 3
Supplementary Figure S1
b
KRAS G12D-NE
IB-21J
KD = 4,810nM
a
KRAS G12D-NE
IB-21G
KD = 641nM
c
KRAS G12D-NE
IIA-15D
KD = 11,600nM
