# Supplementary figures and images for: Discovery of KRB-456, a KRAS G12D Switch-I/II Allosteric Pocket Binder That Inhibits the Growth of Pancreatic Cancer Patient-derived Tumors
Source: Cancer Res Commun. 2023 Dec 28;3(12):2623–39. doi: 10.1158/2767-9764.CRC-23-0222 (PMC10754035; doi:10.1158/2767-9764.CRC-23-0222)

## Slide 1
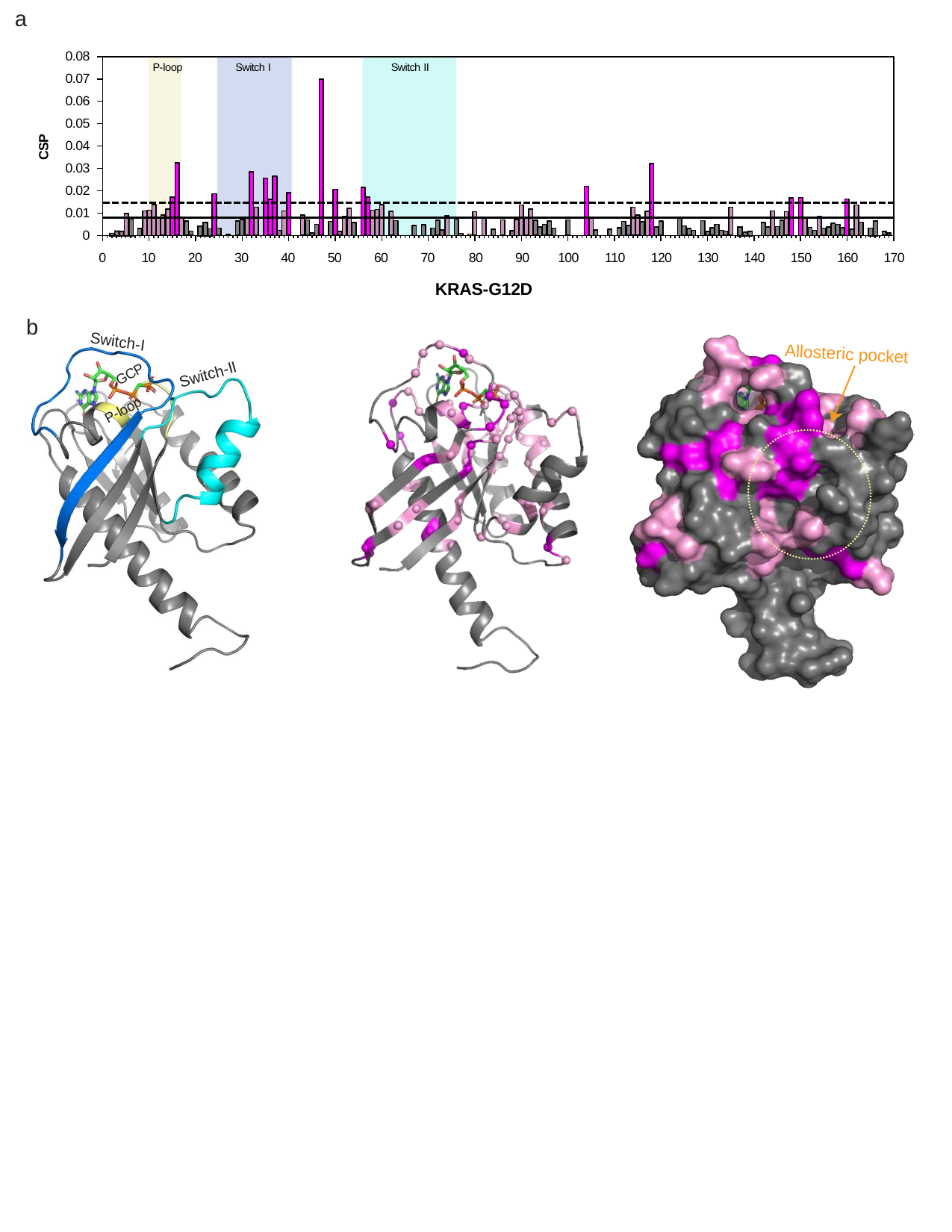

a
0.08
0.07
0.06
0.05
0.04
0.03
0.02
0.01
0
P-loop
Switch I
Switch II
CSP
0
10
20
30
40
50
60
70	80	90	100	110	120	130	140	150	160	170
KRAS-G12D
b
Switch-I
Allosteric pocket
GCP
Switch-II
P-loop

Supplement: Figure S2 — KRB-456 binds to KRAS G12DGCP at a switch-I/II allosteric site. [file crc-23-0222-s02.pptx]

## Slide 1
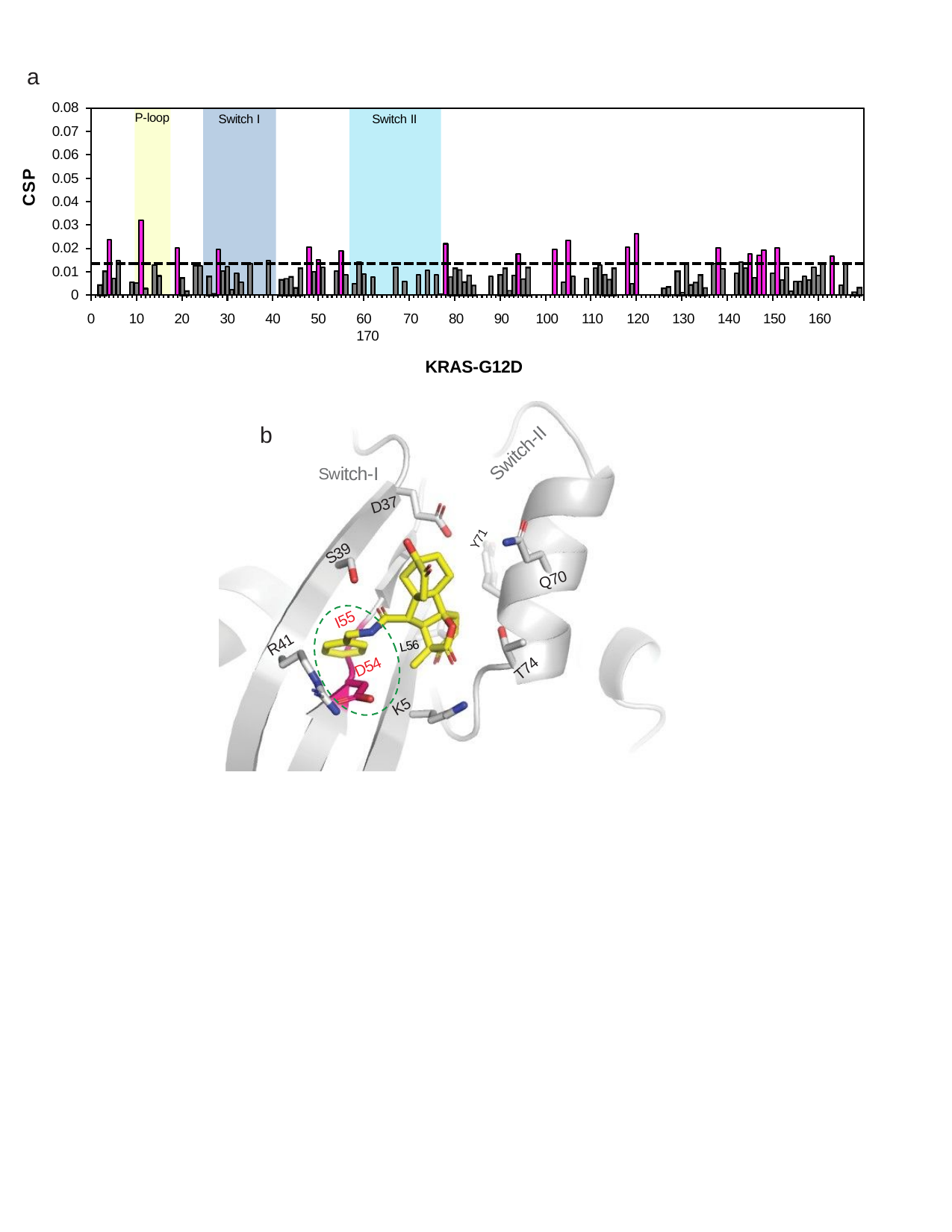

a
0.08
0.07
0.06
0.05
0.04
0.03
0.02
0.01
0
P-loop
Switch I
Switch II
CSP
0
10
20
30
40
50
60	70	80	90	100	110	120	130	140	150	160	170
KRAS-G12D
b
Switch-I
Switch-II
D37
Y71
S39
Q70
I55
R41
L56
D54
T74
K5

Supplement: Figure S4 — IIA-15D shows reduced chemical shift perturbations to KRAS G12DGDP possibly due to steric clash with the KRAS allosteric pocket. [file crc-23-0222-s04.pptx]

## Slide 1
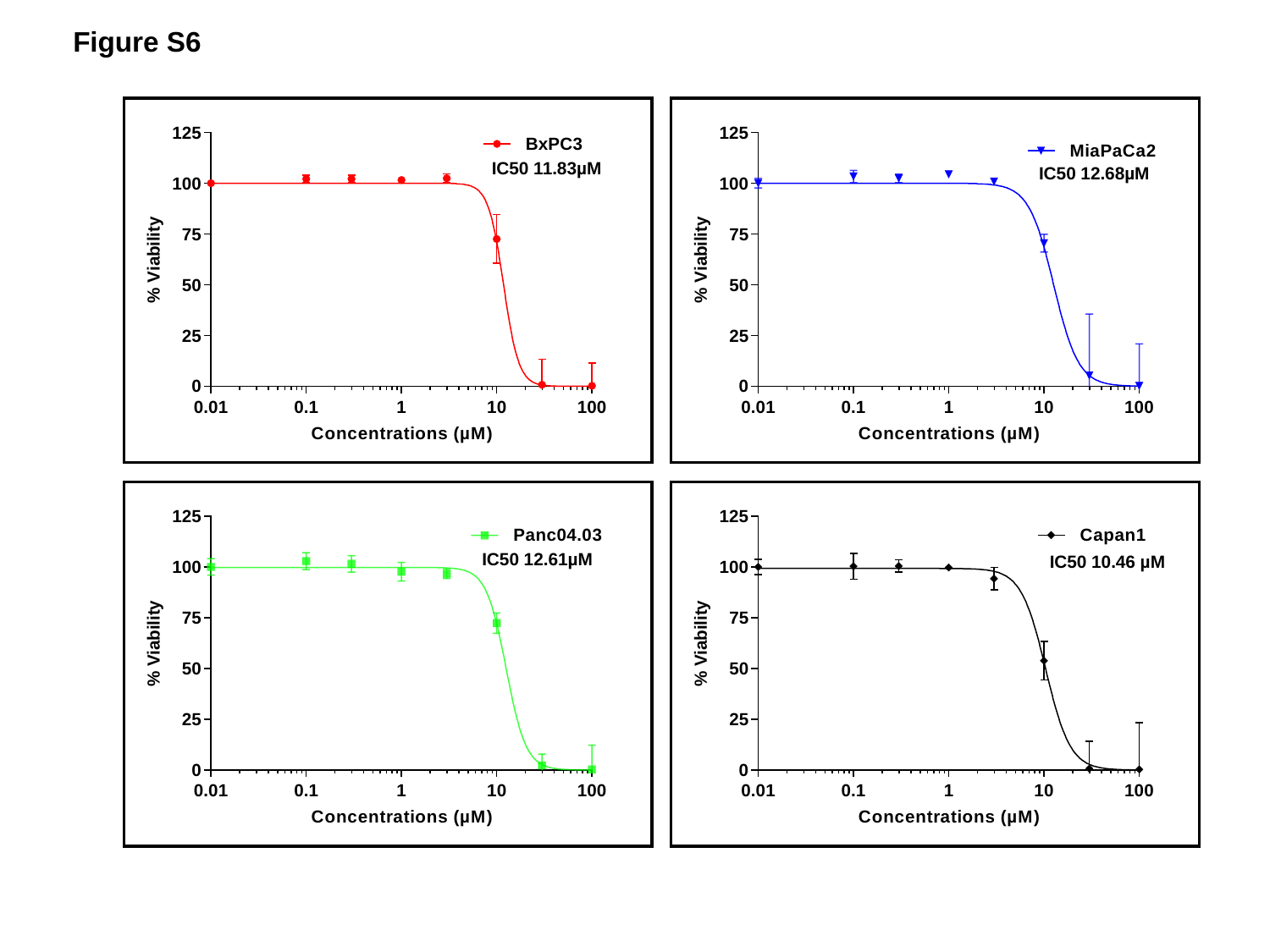

Figure S6
IC50 12.68µM
IC50 11.83µM
IC50 12.61µM
IC50 10.46 µM

Supplement: Figure S6 — Effects of KRB-456 on cell viability in human pancreatic cancer cells that harbor KRAS G12D (Panc0403 cells), KRAS G12V (Capan1 cells), KRAS G12C (MiaPaCa2 cells), and KRAS WT (BxPc3 cells). [file crc-23-0222-s06.pptx]

## Slide 1
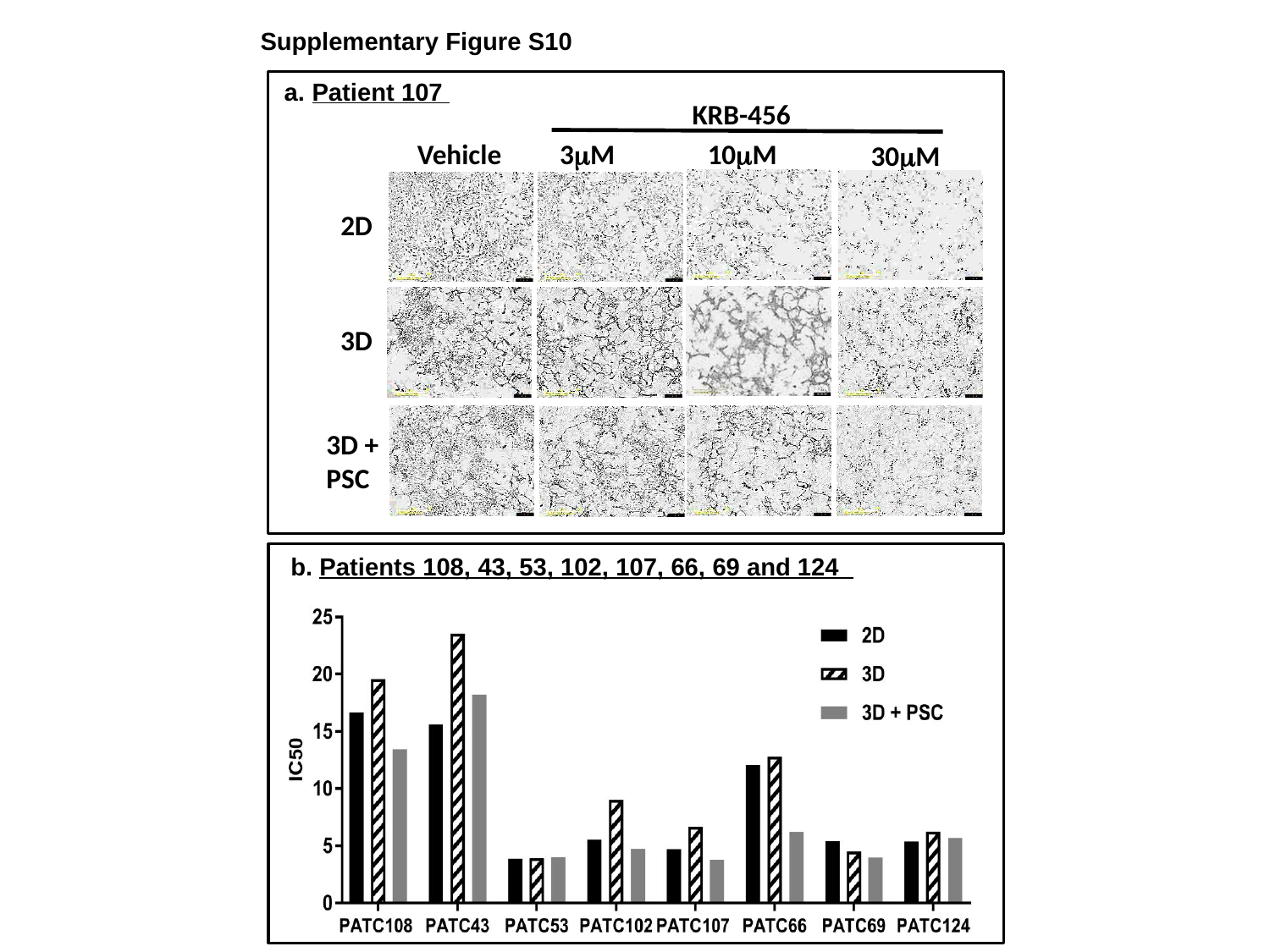

Supplementary Figure S10
a. Patient 107
KRB-456
Vehicle
3mM
10mM
30mM
2D
3D
 3D +
 PSC
b. Patients 108, 43, 53, 102, 107, 66, 69 and 124

Supplement: Figure S10 — KRB-456 inhibits the viability in 2D, 3D, and 3D co-cultures with pancreatic stellate cells (PSCs), of primary and metastatic mt KRAS adenocarcinoma cells derived from 8 pancreatic cancer patients. [file crc-23-0222-s10.pptx]
