# Supplementary material for: Discovery of KRB-456, a KRAS G12D Switch-I/II Allosteric Pocket Binder That Inhibits the Growth of Pancreatic Cancer Patient-derived Tumors
Source: Cancer Res Commun. 2023 Dec 28;3(12):2623–39. doi: 10.1158/2767-9764.CRC-23-0222 (PMC10754035; doi:10.1158/2767-9764.CRC-23-0222)
Supplement: Figure S3 — KRAS switch-I/II allosteric pocket is dynamic. [file crc-23-0222-s03.pptx]

## Slide 1
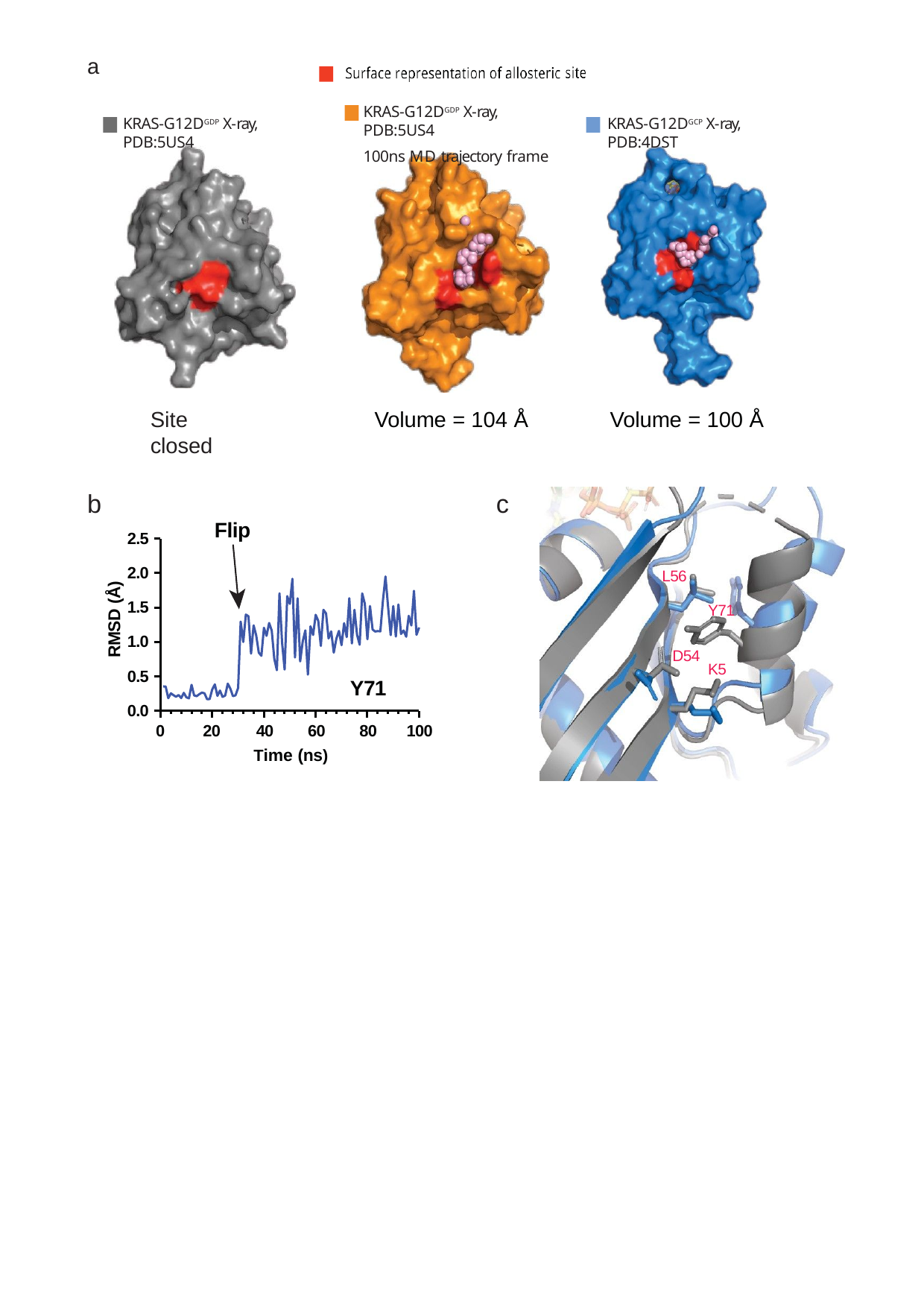

a
KRAS-G12DGDP X-ray, PDB:5US4
100ns MD trajectory frame
KRAS-G12DGDP X-ray, PDB:5US4
KRAS-G12DGCP X-ray, PDB:4DST
Site closed
Volume = 104 Å
Volume = 100 Å
b
c
Flip
2.5
2.0
1.5
L56
RMSD (Å)
Y71
1.0
D54
K5
0.5
0.0
Y71
20	40	60	80	100
Time (ns)
0
