# Supplementary material for: Discovery of KRB-456, a KRAS G12D Switch-I/II Allosteric Pocket Binder That Inhibits the Growth of Pancreatic Cancer Patient-derived Tumors
Source: Cancer Res Commun. 2023 Dec 28;3(12):2623–39. doi: 10.1158/2767-9764.CRC-23-0222 (PMC10754035; doi:10.1158/2767-9764.CRC-23-0222)
Supplement: Figure S5 — BI-2852 competes with KRB-456 for binding to KRAS G12D. [file crc-23-0222-s05.pptx]

## Slide 1
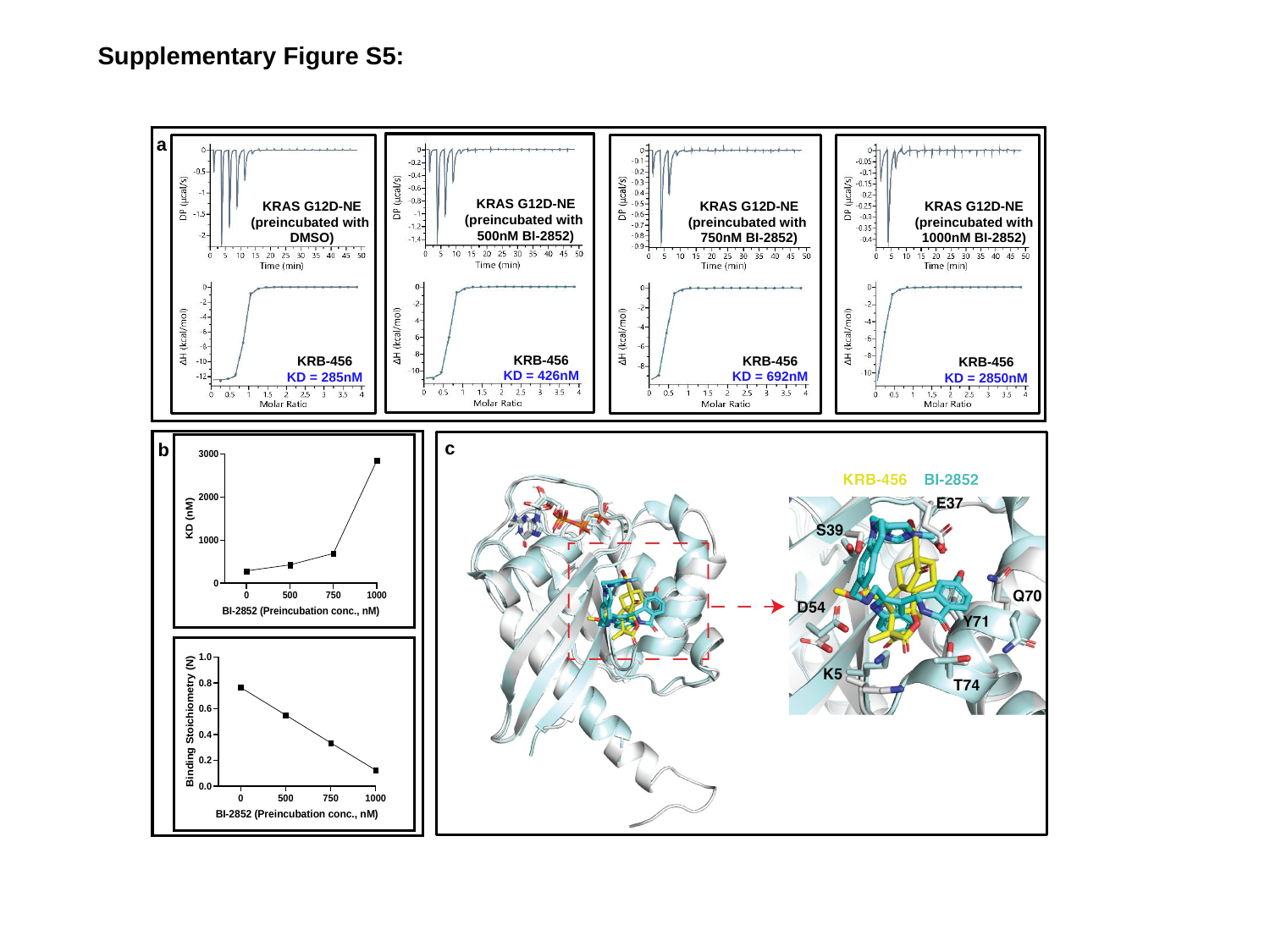

Supplementary Figure S5:
a
KRAS G12D-NE
(preincubated with
500nM BI-2852)
KRB-456
KD = 426nM
KRAS G12D-NE
(preincubated with
DMSO)
KRB-456
KD = 285nM
KRAS G12D-NE
(preincubated with
750nM BI-2852)
KRB-456
KD = 692nM
KRAS G12D-NE
(preincubated with
1000nM BI-2852)
KRB-456
KD = 2850nM
c
b
