# Supplementary material for: Discovery of KRB-456, a KRAS G12D Switch-I/II Allosteric Pocket Binder That Inhibits the Growth of Pancreatic Cancer Patient-derived Tumors
Source: Cancer Res Commun. 2023 Dec 28;3(12):2623–39. doi: 10.1158/2767-9764.CRC-23-0222 (PMC10754035; doi:10.1158/2767-9764.CRC-23-0222)
Supplement: Figure S7 — Effects of KRB-456 on P-MEK and P-ERK levels in human pancreatic cancer Panc0203 and Panc1 cells. [file crc-23-0222-s07.pptx]

## Slide 1
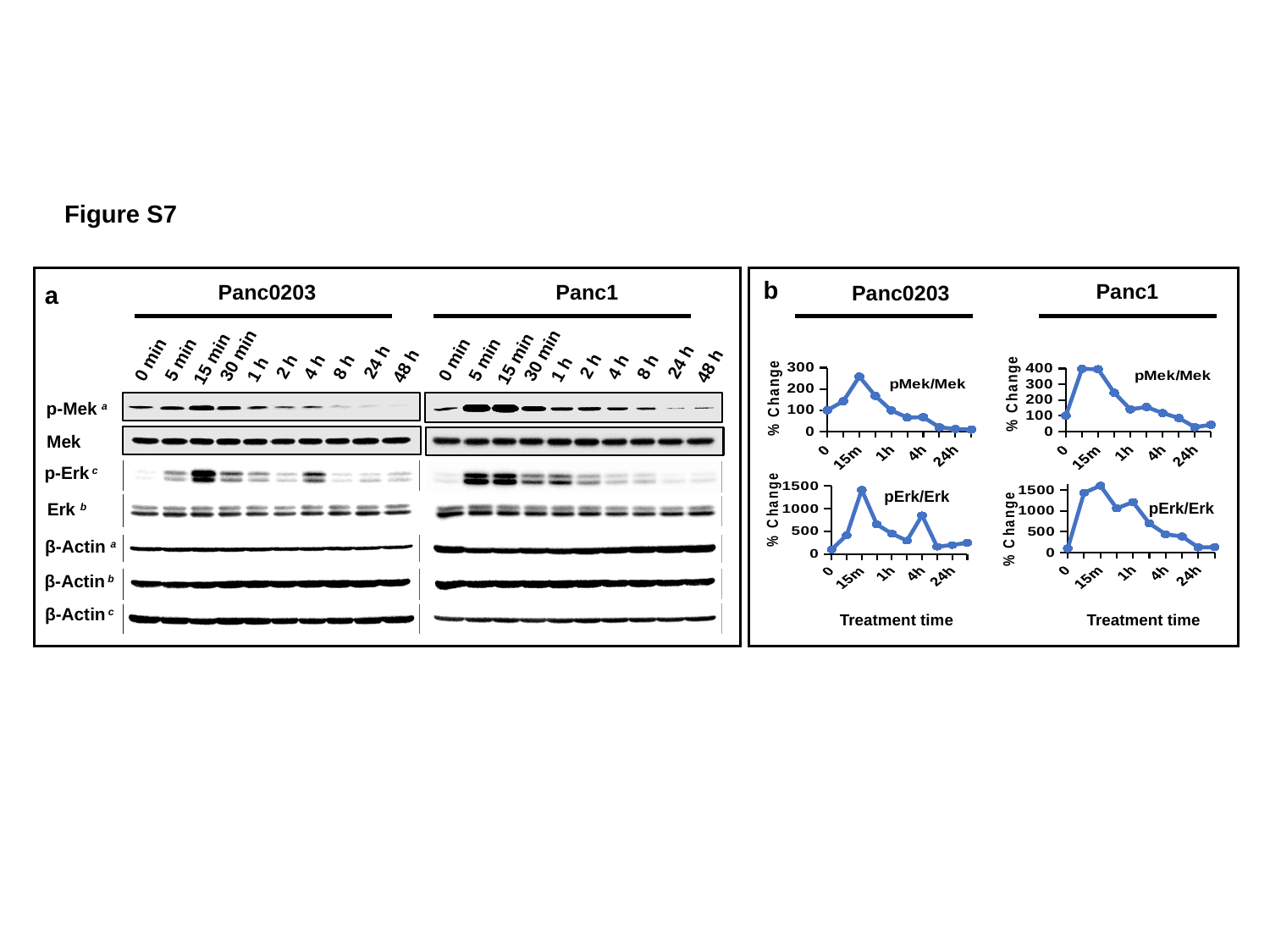

Figure S7
b
Panc1
Panc0203
Panc1
a
Panc0203
30 min
15 min
5 min
0 min
24 h
2 h
8 h
4 h
1 h
48 h
30 min
15 min
5 min
0 min
24 h
2 h
8 h
4 h
1 h
48 h
### Chart: pMek/Mek
| Category | |
|---|---|
| 0 | 100.0 |
| 5m | 142.63601794566998 |
| 15m | 258.30073595500323 |
| 30m | 167.56883681642563 |
| 1h | 99.34501990368125 |
| 2h | 65.89354363755196 |
| 4h | 67.42101845578829 |
| 8h | 20.34261349588712 |
| 24h | 11.701823144537409 |
| 48h | 9.181495282273696 |
### Chart: pMek/Mek
| Category | |
|---|---|
| 0 | 100.0 |
| 5m | 397.5956386294355 |
| 15m | 395.32019151224534 |
| 30m | 245.63797639369537 |
| 1h | 140.4224189157256 |
| 2h | 155.50519380873763 |
| 4h | 116.87038718747193 |
| 8h | 85.77496569068231 |
| 24h | 27.383555962087215 |
| 48h | 42.636787546573416 |p-Mek a
Mek
p-Erk c
### Chart
| Category | |
|---|---|
| 0 | 100.0 |
| 5m | 413.52723514667645 |
| 15m | 1415.6684539083483 |
| 30m | 652.7692772332247 |
| 1h | 449.29896651521364 |
| 2h | 293.9017189129208 |
| 4h | 852.8976397097292 |
| 8h | 161.0592355669976 |
| 24h | 198.27107202674785 |
| 48h | 246.24065464021697 |
### Chart
| Category | |
|---|---|
| 0 | 100.0 |
| 5m | 1434.147746514157 |
| 15m | 1604.1996589136475 |
| 30m | 1061.137122223351 |
| 1h | 1210.4852776350592 |
| 2h | 699.9061577427781 |
| 4h | 438.14473393550077 |
| 8h | 386.7124979887235 |
| 24h | 125.36744113020409 |
| 48h | 129.58408851357666 |pErk/Erk
Erk b
pErk/Erk
β-Actin a
β-Actin b
β-Actin c
Treatment time
Treatment time
