# Supplementary material for: Discovery of KRB-456, a KRAS G12D Switch-I/II Allosteric Pocket Binder That Inhibits the Growth of Pancreatic Cancer Patient-derived Tumors
Source: Cancer Res Commun. 2023 Dec 28;3(12):2623–39. doi: 10.1158/2767-9764.CRC-23-0222 (PMC10754035; doi:10.1158/2767-9764.CRC-23-0222)
Supplement: Figure S8 — KRB-456 inhibits the growth in vivo of orthotopic mt KRAS tumors derived from pancreatic cancer patients. [file crc-23-0222-s08.pptx]

## Slide 1
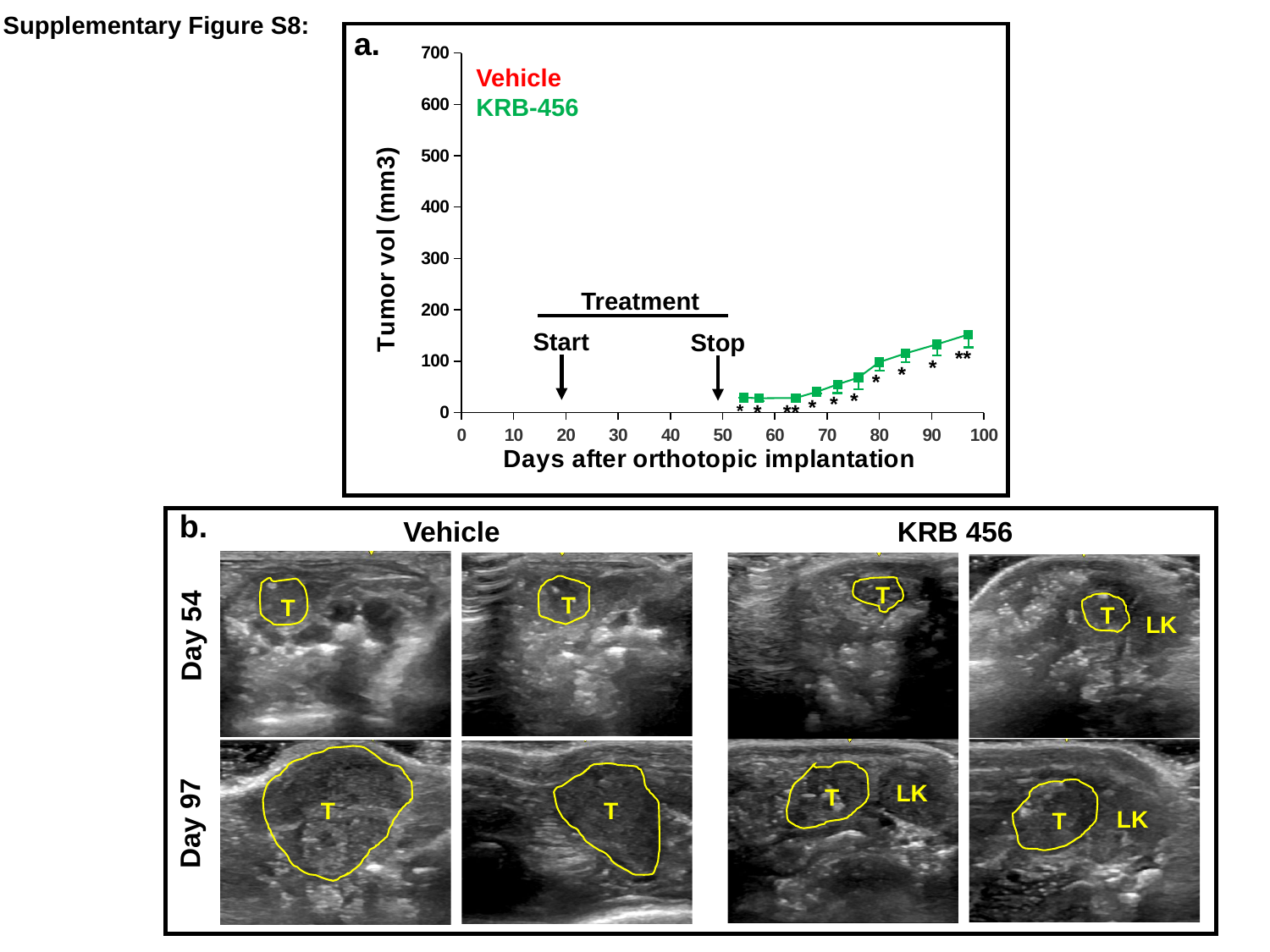

Supplementary Figure S8:
a.
### Chart
| Category | Vehicle (n=7) | 5mpk KRB456 (n=7) |
|---|---|---|Vehicle
KRB-456
Start
**
*
*
*
*
*
*
**
*
*
Stop
Treatment
b.
Vehicle
KRB 456
T
T
T
T
LK
Day 54
LK
T
T
T
Day 97
LK
T
