# Supplementary material for: Discovery of KRB-456, a KRAS G12D Switch-I/II Allosteric Pocket Binder That Inhibits the Growth of Pancreatic Cancer Patient-derived Tumors
Source: Cancer Res Commun. 2023 Dec 28;3(12):2623–39. doi: 10.1158/2767-9764.CRC-23-0222 (PMC10754035; doi:10.1158/2767-9764.CRC-23-0222)
Supplement: Figure S9 — Treatment of mice with KRB-456 inhibits P-MEK, P-AKT and P-S6 levels and induces apoptosis in KRAS G12D PDXs from pancreatic cancer patients. [file crc-23-0222-s09.pptx]

## Slide 1
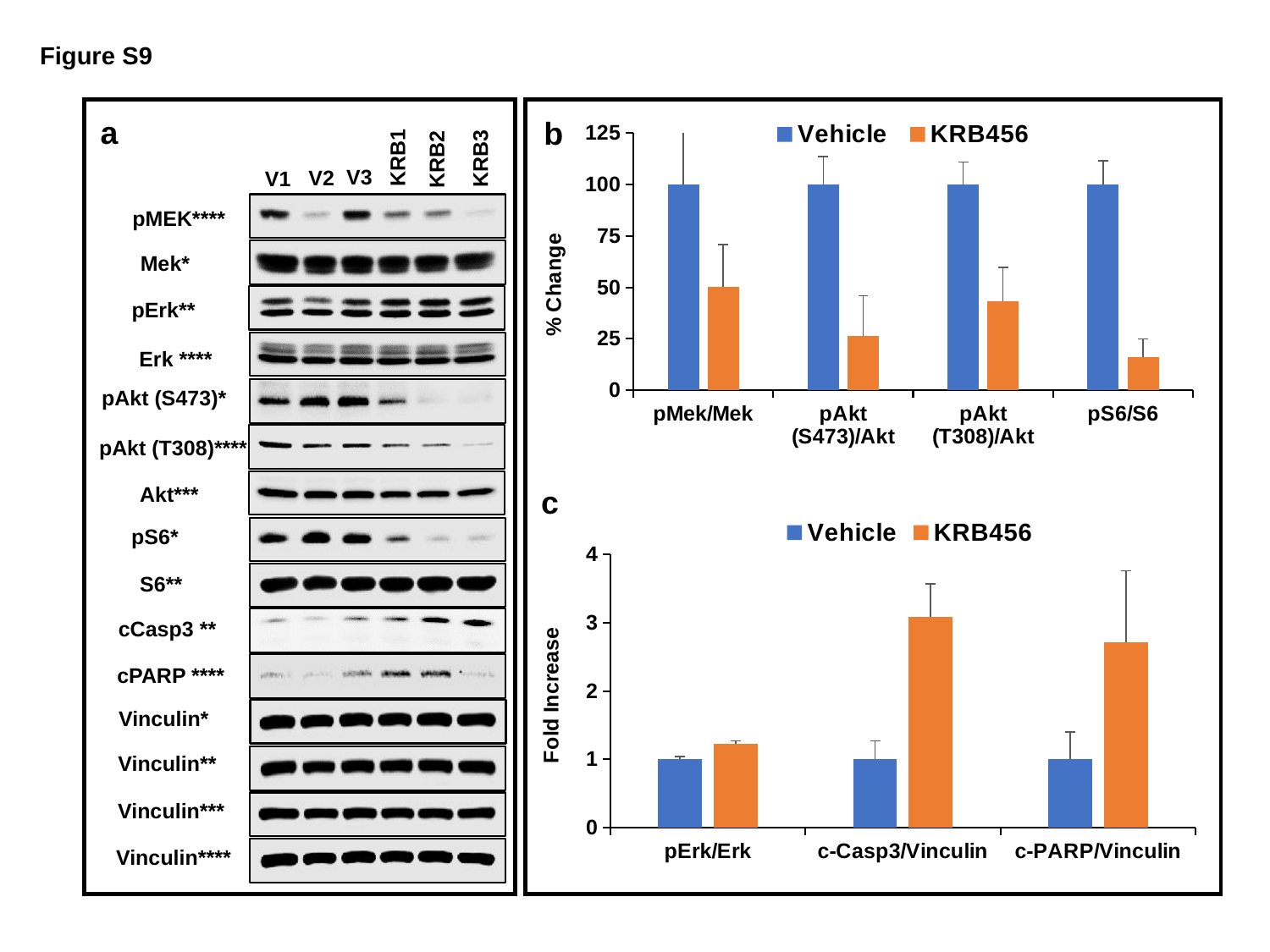

Figure S9
### Chart
| Category | Vehicle | KRB456 |
|---|---|---|
| pMek/Mek | 100.0 | 50.06 |
| pAkt (S473)/Akt | 100.0 | 26.55 |
| pAkt (T308)/Akt | 100.0 | 43.34 |
| pS6/S6 | 100.0 | 16.06 | KRB1
 KRB3
 KRB2
 V3
 V2
 V1
pMEK****
Mek*
pErk**
Erk ****
pAkt (S473)*
pAkt (T308)****
Akt***
### Chart
| Category | Vehicle | KRB456 |
|---|---|---|
| pErk/Erk | 1.0 | 1.23 |
| c-Casp3/Vinculin | 1.0 | 3.09 |
| c-PARP/Vinculin | 1.0 | 2.72 |pS6*
S6**
cCasp3 **
cPARP ****
Vinculin*
Vinculin**
Vinculin***
Vinculin****
a
b
c
