# Supplementary material for: Discovery of KRB-456, a KRAS G12D Switch-I/II Allosteric Pocket Binder That Inhibits the Growth of Pancreatic Cancer Patient-derived Tumors
Source: Cancer Res Commun. 2023 Dec 28;3(12):2623–39. doi: 10.1158/2767-9764.CRC-23-0222 (PMC10754035; doi:10.1158/2767-9764.CRC-23-0222)
Supplement: Supplementary Figure Legends — Supplemental Figure Legends [file crc-23-0222-s11.doc]

**Supplementary Figure Legends**

**Figure S1: KRB-456 analogs IB-21G, IB-21J and IIA-15D binds KRAS G12D with lower affinity.** Isothermal titration calorimetry (ITC) assays were performed using 2 mM IB-21G, IB-21J or IIA-15D in the ITC injector and 100 µM protein in the ITC sample cell as described in Methods.

**Figure S2.** **KRB-456 binds to KRAS G12D^GCP^ at a switch-I/II allosteric site. (a)** Measured chemical shift perturbations (CSPs) of ^15^N-labeled KRAS G12D^GCP^ upon KRB-456 titration up to a ratio of 1:4 KRAS G12D^GCP^: KRB-456 are plotted as a function of KRAS residue number. Residues with chemical shift ranges over the significance threshold or 1.5 times the threshold are colored pink and magenta, respectively. Residues associated with the P-loop, the switch-I and switch-II structural motifs are indicated with yellow, blue and cyan shading, respectively. Data are representative of 3 independent experiments. **(b)** Ribbon representation of KRAS G12D^GCP^ structure (gray; PDB: 4DST) indicating the key structural motifs and mapping of residues undergoing significant CSP onto the ribbon and surface representation of KRAS G12D, as in a, in pink and magenta. Several residues with significant and the highest CSPs concentrate around the allosteric pocket (dotted circle line) that is formed in the switch-I/II helix region as with the KRAS G12D^GDP^ conformation.

**Figure S3.** **KRAS switch-I/II allosteric pocket is dynamic. (a)** Analysis of MD trajectory of KRAS G12D^GDP^ (PDB 5U54, left panel) reveals opening of the switch-I/II allosteric pocket after 100 ns of simulation (middle panel). Analysis of the allosteric pocket (key residues in red color) using Sitemap (Schrodinger LLC) of the KRAS G12D^GDP^ structure at 100 ns and KRAS G12D^GCP^ (PDB 4DST, right panel), gives comparable allosteric pocket volumes (pink spheres). **(b)** RMSD analysis during MD simulation of KRAS G12D^GDP^ (PDB 5U54) of the pocket residue Y71 indicates a conformational flip at around 30 ns. **(c)** Structural superposition of KRAS G12D^GCP^ (PDB 4DST, blue) and KRAS G12D^GDP^ (100 ns snapshot of MD simulation, gray) illustrating the conformational flip of Y71, which largely results to the opening of the allosteric site.

**Figure S4. IIA-15D shows reduced chemical shift perturbations to KRAS G12D^GDP^ possibly due to steric clash with the KRAS allosteric pocket. (a)** Measured chemical shift perturbations (CSP) of ^15^N-labeled KRAS G12D^GDP^ upon IIA-15D titration up to a ratio of 1:4 KRAS G12D^GDP^: IIA-15D are plotted as a function of KRAS residue number. Residues with chemical shift ranges over 1.5 times the threshold are colored magenta. Data are representative of 3 independent experiments. **(b)** Close-up view of the KRAS G12D allosteric binding site bound to the hypothetical structure of IIA-15D (yellow) based on KRB-456 docked conformation. Analysis of the docked pose of IIA-15D suggests a possible steric clash (green dotted circle line) of the phenyl ring of IIA-15D with the main chain of residues D54 and I55, generating a less stable binding for IIA-15D.

**Figure S5. BI-2852 competes with KRB-456 for binding to KRAS G12D.** KRAS G12D was preincubated for 15 minutes with BI-2852 (0, 500, 750 and 1000 nM) prior to determining KRB-456 Kds using Isothermal Titration Calorimetry (ITC) as described in Methods. **(a)** Binding affinity (Kd) of KRB-456 for KRAS G12D was 285 nM, 426nM, 692nM and 2850nM when KRAS G12D was preincubated with 0, 500nM, 750nM and 1000nM BI-2852, respectively. **(b)** Graphical representation of effects of BI-2852 pre-incubation on KRB-456 binding affinity (Kd) and binding stoichiometry (N). **(c)** Structural superposition of the crystal structure KRAS G12D GCP bound to BI-2852 (PDB ID: 6GJ8, cyan) and KRB-456 docked structure of KRAS G12D GCP (gray) illustrating the overlapping binding of BI-2852 (cyan sticks) and KRB-456 (yellow sticks) at the allosteric site. (Inset) Zoomed view at the KRAS G12D GCP allosteric site showing BI-2852 (cyan sticks), KRB-456 (yellow sticks) and allosteric site residues (cyan or gray sticks).

**Figure S6: Effects of KRB-456 on cell viability in human pancreatic cancer cells that harbor KRAS G12D (Panc0403 cells), KRAS G12V (Capan1 cells), KRAS G12C ( MiaPaCa2 cells), and KRAS WT (BxPc3 cells).** The human pancreatic cancer cell lines were treated with the indicated concentrations of KRB-456 for 72 hours and processed for viability assays as described under Methods.

**Figure S7: Effects of KRB-456 on P-MEK and P-ERK levels in human pancreatic cancer Panc0203 and Panc1 cells.**  **(a)** Human pancreatic cancer cell lines Panc0203 and Panc1 were treated with KRB-456 for the indicated time points and processed for western blotting as described in Methods. **(b)** Quantification of the western blots.

**Figure S8:** **KRB-456 inhibits the growth *in vivo* of orthotopic mt KRAS tumors derived from pancreatic cancer patients.** Tumor biopsies from pancreatic cancer patient G160 were prepared and implanted orthotopically into NSG mice as described in Methods. On day 20 after implantation, mice were treated i.p. daily for 30 days with vehicle or 5mpk KRB-456, and monitored for an additional 47 days without treatment. Tumors were monitored by ultrasound starting on day 40, but only became big enough to be measurable starting on day 54, and tumor measurements continued until day 97 after implantation. Yellow encircled area designates tumor (T). LK designates Left Kidney. P values determined by Student’s t-test (*P < 0.05; **P < 0.01). Error bars represent standard error.

**Figure S9. Treatment of mice with KRB-456 inhibits P-MEK, P-AKT and P-S6 levels and induces apoptosis in KRAS G12D PDXs from pancreatic cancer patients. (a)** Tumor biopsies from patient G166 were s.c. implanted into NSG mice as described in Methods, and when the average tumor volume reached 200 mm^3^, the mice were treated either with vehicle (mice V1, V2, V3) or 5 mpk KRB-456 (mice KRB1, KRB2, KRB3) as described in Methods. Two hours after treatment, tumors were harvested and lysates were processed for western blotting as described in Methods. * , **, ***, and **** designate blots from different gels.  **(b)** and **(c)** Quantification of the western blots. Error bars represent standard error.

**Figure S10.** **KRB-456 inhibits the viability in 2D, 3D, and 3D co-cultures with pancreatic stellate cells (PSCs), of primary and metastatic mt KRAS adenocarcinoma cells derived from 8 pancreatic cancer patients.** Cells derived from patients 43, 53, 66. 69,102, 107, 108 and 124 were treated for 72 hours with KRB-456**. (a)** representative live-cell images data from cells derived from patient 107**. (b)** IC50 values in µM of the effects of KRB-456 on the viability (determined by CellTiter-Glo luminescence) of cells derived from 8 pancreatic cancer patients.
