# Supplementary material for: Discovery of KRB-456, a KRAS G12D Switch-I/II Allosteric Pocket Binder That Inhibits the Growth of Pancreatic Cancer Patient-derived Tumors
Source: Cancer Res Commun. 2023 Dec 28;3(12):2623–39. doi: 10.1158/2767-9764.CRC-23-0222 (PMC10754035; doi:10.1158/2767-9764.CRC-23-0222)
Supplement: Supplementary Methods — Supplemental Methods [file crc-23-0222-s12.docx]

**Supplemental Methods**

**Expression and purification of RAS proteins:** Human His-tagged KRAS4B (1-169) G12D, G12V and G12C were custom synthesized and cloned into pET-28a(+)-TEV vector to generate pET-28a(+)-6His-KRAS (1-169) plasmids by GenScript USA Inc., Piscataway, NJ. The proteins were expressed and purified as described below. The full length biotinylated human KRAS4B G12D (His6-MBP-tev-Avi-Hs.KRAS4b G12D (2-188) protein was obtained from Dr Dominic Esposito and Dr. William Gillette from the NCI RAS Initiative at the Frederick National Laboratory. Avi-KRAS4b (2-188) G12D was cloned, expression and purified as described previously (1,2).

**Expression and purification of KRAS proteins:** BL21 star (DE3) competent cells were transformed with pET-28a(+)-6His-KRAS (1-169) plasmids from GenScript, and the resulting colonies were picked under kanamycin selection. The colonies were grown in LB broth and induced with 0.5mM IPTG for 4 hours when OD reached 0.6-0.8. Bacteria pellets were lysed in 25mM Tris HCL (pH7.8) buffer containing 300mM NaCl, 5mM MgCl2, 10mM Imidazole, 5% Glycerol and 3mM β-mercaptoethanol. Membrane filter-cleared lysate supernatants were incubated with HisPur Ni-NTA resin (Thermo scientific) in an oscillating platform at 4°C for 50 minutes. Econo disposable column (Bio-Rad) was used for washing the beads 3 times with 25mM Tris HCl (pH7.8), 300mM NaCl, 5mM MgCl2, 5% Glycerol buffer with 10mM, 20mM and 30mM Imidazole, respectively. Elution was carried with 25mM Tris HCl (pH7.8), 300mM NaCl, 5mM MgCl2, 5% Glycerol and 500mM Imidazole buffer. After confirming the eluted fraction purity on SDS-PAGE gel, proteins fractions were further purified using HiLoad 16/600 superdex 75 PG column in AKTA system. Protein was eluted in 25mM Tris HCL (pH7.8), 150mM NaCl, 5mM MgCl2 and 0.5mM DTT. KRAS protein aliquots were stored in -80°C.

**Expression and purification of GST-RBD:** The Raf1-GST-RBD in pGEX-2T vector in DH5α cells was obtained from Addgene (Raf1 GST RBD 1-149, Plasmid catalog # 13338). GST-RBD plasmid DNA was transformed in BL21*(DE3) bacteria, streaked on agar plate to obtain the colonies . A distinct colony was picked and grown in ampicillin selection media to OD600 of around 0.6-0.8 before induction with IPTG (0.5 mM). Bacterial pellet was resuspended with lysis buffer (50 mM Tris HCL (pH 7.5), 150 mM NaCl, 0.5% Triton X-100, 5 mM MgCl_2_, 1 mM DTT, 10% Glycerol, 3 mM β-mercaptoethanol) and homogenized. The homogenized lysate was centrifuged at 14000 *rpm* at 4˚C for 40 min and the resulting supernatant was collected and filtered. The filtered supernatant was incubated with glutathione agarose (Pierce^TM^) beads (Thermo Scientific, USA) at 4˚C for 60 min. After incubation, beads and lysate suspension was passed through Bio-Rad Econo column. The beads were washed using lysis buffer (as described above), and the GST-RBD eluted with elution buffer (50 mM Tris HCL (pH 7.5), 150 mM NaCl, 0.5% Triton X-100, 5 mM MgCl_2_, 1 mM DTT, 10% Glycerol, 10 mM Glutathione). Eluted GST-RBD protein fractions were buffer exchanged into 25 mM Tris (pH 7.5), 150 mM NaCl, 0.5 mM DTT and 5 mM MgCl_2_ buffer and stored at -80°C.

**Nucleotide Exchange:**

GCP and NH-GDP nucleotide exchange for ITC: KRAS G12D protein in 25 mM Tris HCL (pH 8.0), 1 mM Calcium Chloride Dihydrate and 200 mM (NH4)2SO4 buffer was added to alkaline phosphatase beads (Sigma, USA) (2U per mg of KRAS) and to non-hydrolysable nucleotide analogs, GCP (β,γ-Methyleneguanosine 5′-triphosphate sodium salt, Sigma, USA) or GDP-NH (Guanosine 5′-[β-thio]diphosphate trilithium salt, Sigma, USA) in molar ratio of 10:1 (Nucleotide:KRAS). The reaction mixture was incubated with gentle rotation for 14-18 hrs at 4˚C. After incubation, the mixture was centrifuged at 1500 *g*, 4°C to remove the alkaline phosphatase beads. MgCl_2_ (20 mM) and an additional amount of GCP or NH-GDP in molar ratio of 5:1 (Nucleotide:KRAS) were added, and incubated at room temperature for 1 hr. After incubation, nucleotide exchanged protein was desalted and buffer exchanged at 4˚C using PD-10 column (Catalog # 17085101, GE Healthcare, USA), into a final buffer containing 25 mM Tris HCL (pH 7.5), 150 mM NaCl, 5 mM MgCl_2_ and 0.5 mM DTT. Buffer exchanged protein was concentrated using Amicon ultra centrifugal filter-10K cut off (Catalog # UFC501024, MilliporeSigma, USA). The protein concentration was measured using Pierce Rapid GOLD BCA Protein Assay Kit (Thermo Scientific, USA) and aliquots were stored in -80°C.

GTP and GDP nucleotide exchange for GST-RBD pull down: KRAS protein was exchanged with GTP (Sigma, USA) and GDP (Sigma, USA) using EDTA- loading procedure. The RAS proteins (235 µM final) were incubated with ~40-fold molar excess of EDTA (10 mM final), and ~45-fold molar excess of either GDP or GTP for 90 min at 37°C. After incubation, the sample was kept on ice for 10 minutes before incubating with ice-cooled 1M MgCl_2_ (65 mM final) for 20 minutes to stop the reaction. To remove excess unbound nucleotide, EDTA and magnesium, the proteins were re-buffered using PD10 columns (GE Life Sciences) into (25 mM Tris (pH 7.5), 150 mM NaCl, 0.5 mM DTT and 5 mM MgCl_2_) buffer and concentrated using Millipore spin ultrafiltration devices (10,000 Da cut off, 4,000 x g). The protein concentration was measured using Pierce Rapid GOLD BCA Protein Assay Kit (Thermo Scientific, USA) and aliquots were stored in -80°C**.**

**Molecular docking and molecular dynamics calculations:** Docking of KRB-456 and IIA-15D on KRAS-G12D^GCP^ structure (PDB:4DST) was performed using induced-fit docking (IFD, Schrodinger, LLC) with extra precision (XP). Guided by the NMR data, the binding site was centered around residues K5, V7, D54, D56, L57, E37, S39, Y71 and T74. KRB-456 and IIA-15D was converted to 3D all atom structure using LIGPREP (Schrodinger, LLC) and assigned partial charges with EPIK (Schrodinger, LLC). The best (lower energy) pose from IFD of KRB-456 was subjected to 3 independent 80 nsec MD simulations using Desmond (Schrodinger, LLC). To assess the dynamics of KRAS G12D in the GDP and GTP states, 3 independent 100 ns MD simulations were performed on KRAS G12D/GDP (PDB 5US4) and KARSG12D/GCP (pdb 4DST) structures. All MD runs were performed in a truncated TIP3P water box using OPLS3 force field, 300K, and constant pressure of 1.0325 bar. Analysis of trajectories was performed with Desmond simulation event analysis tools within Maestro (Schrodinger, LLC). Ligand and protein RMSF was calculated using formula RMSF= √(1/t SUM ( ( r_i_’(t) – r_i_(t_0_) ) ^2 ) where t is the trajectory time, r_i_ is the position of atom i in the start of simulation (t_0_) and r_i_’ is the position of atom i at time t after superposition on first frame at t_0_. Ligand interaction diagrams were prepared using Maestro (Schrodinger, LLC) and pose figures using PyMOL (Schrodinger, LLC).

**References**

1. Tran TH, Chan AH, Young LC, Bindu L, Neale C, Messing S*, et al.* KRAS interaction with RAF1 RAS-binding domain and cysteine-rich domain provides insights into RAS-mediated RAF activation. Nat Commun **2021**;12:1176

2. Kopra K, Vuorinen E, Abreu-Blanco M, Wang Q, Eskonen V, Gillette W*, et al.* Homogeneous dual-parametric-coupled assay for simultaneous nucleotide exchange and KRAS/RAF-RBD interaction monitoring. Anal Chem **2020**;92:4971-9
