# Supplementary material for: Discovery of KRB-456, a KRAS G12D Switch-I/II Allosteric Pocket Binder That Inhibits the Growth of Pancreatic Cancer Patient-derived Tumors
Source: Cancer Res Commun. 2023 Dec 28;3(12):2623–39. doi: 10.1158/2767-9764.CRC-23-0222 (PMC10754035; doi:10.1158/2767-9764.CRC-23-0222)
Supplement: Supplementary Table S1 — Structure-activity relationship studies of the inhibition of KRAS G12D/GST-RBD binding. [file crc-23-0222-s13.docx]

**Supplementary Table S1: Structure-activity relationship studies of the inhibition of KRAS G12D/GST-RBD binding.** The top 4 confirmed hits from Figure 1 were all derivatives of gibberellic acid and were evaluated for their potency to inhibit KRAS G12D/GST-RBD binding by GST pull-down assays (see Figure 2). The IC50 values in µM were determined from the concentration-response experiments of Figure 2.
